# Selective extraction of lithium over alkali and alkaline earth ions by synergistic solvent extraction†

**DOI:** 10.1039/d4gc04760e

**Published:** 2024-12-20

**Authors:** Stijn Raiguel, Laura Van Bogaert, Tim Balcaen, Koen Binnemans

**Affiliations:** a KU Leuven, Department of Chemistry Celestijnenlaan 200F P.O. box 2404 B-3001 Leuven Belgium stijn.raiguel@kuleuven.be

## Abstract

Direct lithium extraction (DLE) from natural surface and geothermal brines is very challenging due to the low ratio of lithium to other metals, and the lack of suitable materials that bind lithium with sufficiently high selectivity. In this paper, a synergistic solvent extraction system is described that comprises a liquid ion exchanger (saponified bis(2-ethylhexyl)dithiophosphoric acid) and a lithium-selective ligand (2,9-dibutyl-1,10-phenanthroline) in an aliphatic diluent. The extraction mechanism was investigated and was confirmed to involve the binding of lithium to the selective ligand, while the liquid ion exchanger facilitates the transfer of metal ions from the aqueous to the organic phase. The variables influencing the selectivity for lithium were also determined. The selectivity improved greatly in highly concentrated salt solutions with low concentrations of lithium, rendering the process ideal for the sequestration of lithium from natural brines. Stripping could be achieved with stoichiometric amounts of hydrochloric acid. Applying the system to a synthetic geothermal brine, an extraction percentage of 68% was obtained in a single stage, with separation factors of 620 ± 20 for lithium over sodium, 3100 ± 200 for lithium over potassium, 596 ± 9 for lithium over magnesium and 2290 ± 80 for lithium over calcium.

## Abstract 2

A synergistic extraction system comprising a dithiophosphoric acid and a 1,10-phenanthroline derivative selectively extracts lithium from synthetic brines. The mechanism, thermodynamics and the variables influencing the selectivity are discussed.

## Graphical abstract

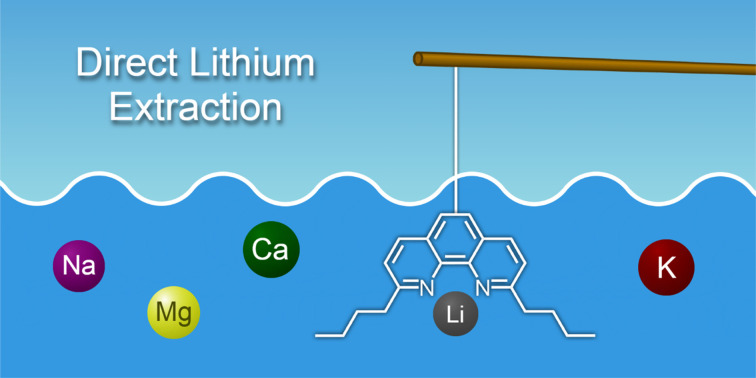

**Green foundation**

1. This work proposes a new method to extract lithium from continental and geothermal brines using solvent extraction. The state-of-the-art method is plagued by high water consumption in arid regions, a large plant footprint, and substantial reagent use and waste production.

2. Compared to the state of the art process for treatment of lithium brines, the proposed DLE process obviates the need for brine evaporation, reducing both water consumption and the footprint of the plant. Furthermore, the alkaline earth elements no longer need to be precipitated, drastically curbing reagent use and waste production. Hence, the proposed process offers opportunities to significantly improve the sustainability of brine lithium production.

3. This work provides the fundamental basis of a process. The mechanisms are investigated and the fundamental thermodynamic and equilibrium properties are established. However, optimization and long-term continuous tests on real brines are prerequisite for commercialization of a hydrometallurgical process. These tests lie beyond the scope of the current research.

## Introduction

Continental brines (in *salars*) constitute a major commercial source of lithium, and hold the majority of the world's lithium reserves.^1^ The state-of-the-art processing route entails a slow preconcentration of the natural brine, achieved through solar evaporation. Subsequently, borates are often removed by solvent extraction of boric acid with iso-octanol, while magnesium and sulfates are usually precipitated using lime. Lithium is finally precipitated as its carbonate salt by addition of soda ash, and further downstream purification steps are applied if battery-grate lithium carbonate is required.^2^ The chief disadvantages of brine-sourced lithium are the lengthy production times (up to 18 months) and the large water consumption resulting from the evaporation step.^3,4^ Furthermore, nearly 50% of the available lithium is not recovered, and it is impossible to obtain LiOH·H_2_O directly.^3,5^ The latter lithium salt is required for the production of Ni-rich (and Co-lean) NMC cathode-active materials, and its synthesis from the intermediate Li_2_CO_3_ entails a significant additional production cost.^6,7^ Other brine sources, such as geothermal brines and oil field brines, cannot be treated by means of the evaporation process, due in part to the lithium concentration being so low as to render the process commercially unviable.^8^ Direct lithium extraction (DLE), *i.e.* the selective sequestration of lithium from brines, is an attractive concept as it avoids the aforementioned issues with the conventional extraction process.^9,10^ A DLE process would bypass the need for the lengthy evaporation step, while also obviating the chemical cost and waste production associated with the precipitation step. An ideal DLE process would allow the depleted brine to be re-injected into the *salar*, avoiding the environmental damage associated with dropping ground water levels in arid areas. Concurrently, lithium would not have to be precipitated as the carbonate, and could hence be directly converted to the coveted hydroxide, for instance *via* electrodialysis.^2^

Few methods exist to directly remove lithium from brine solutions. Adsorption and ion exchange have seen limited commercialization, but produce highly dilute lithium solutions which must first be concentrated by boiling to allow precipitation of the carbonate, rendering the process energy-intensive.^11,12^ Furthermore, most adsorbents suffer from low capacities for lithium and have a limited chemical stability.^11^ A solvent extraction process, on the other hand, would allow the removal of lithium from fresh brine by simply contacting the brine with a solvent, and could be used to concentrate the lithium solution by stripping with small volumes of a concentrated stripping liquor.

A number of solvent extraction processes for lithium have been investigated.^13–15^ One such solvent extraction process for lithium is known as LiSX™ and was developed by the Italian company TENOVA. The process is rapid and cost-effective compared to standard methods, as no evaporation and no boron solvent extraction are required. However, the extractant used in the process is not selective for lithium over calcium and magnesium, and these elements must first be removed before the extraction can take place. Hence, this technology cannot be considered as a sound DLE approach.^3,16^

**Fig. 1:**
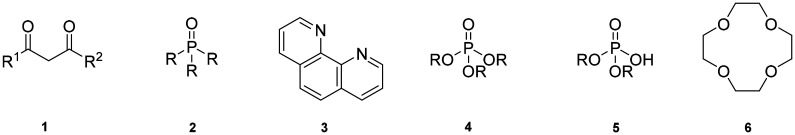
Structures of reported extractants for lithium solvent extraction. 1: β-Diketone. 2: Trialkylphosphine oxide. 3: 1,10-Phenanthroline. 4: Trialkyl phosphate. 5: Dialkylphosphoric acid. 6: Crown ether.

**Fig. 2:**
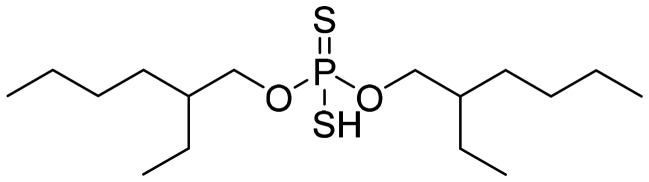
Structure of bis(2-ethylhexyl)dithiophosphoric acid (D2EHDTPA).

**Fig. 3:**
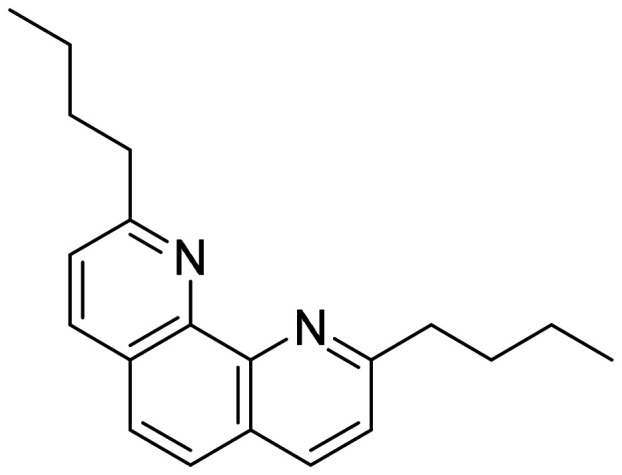
Structure of 2,9-dibutyl-1,10-phenanthroline (BuPhen).

**Fig. 4:**
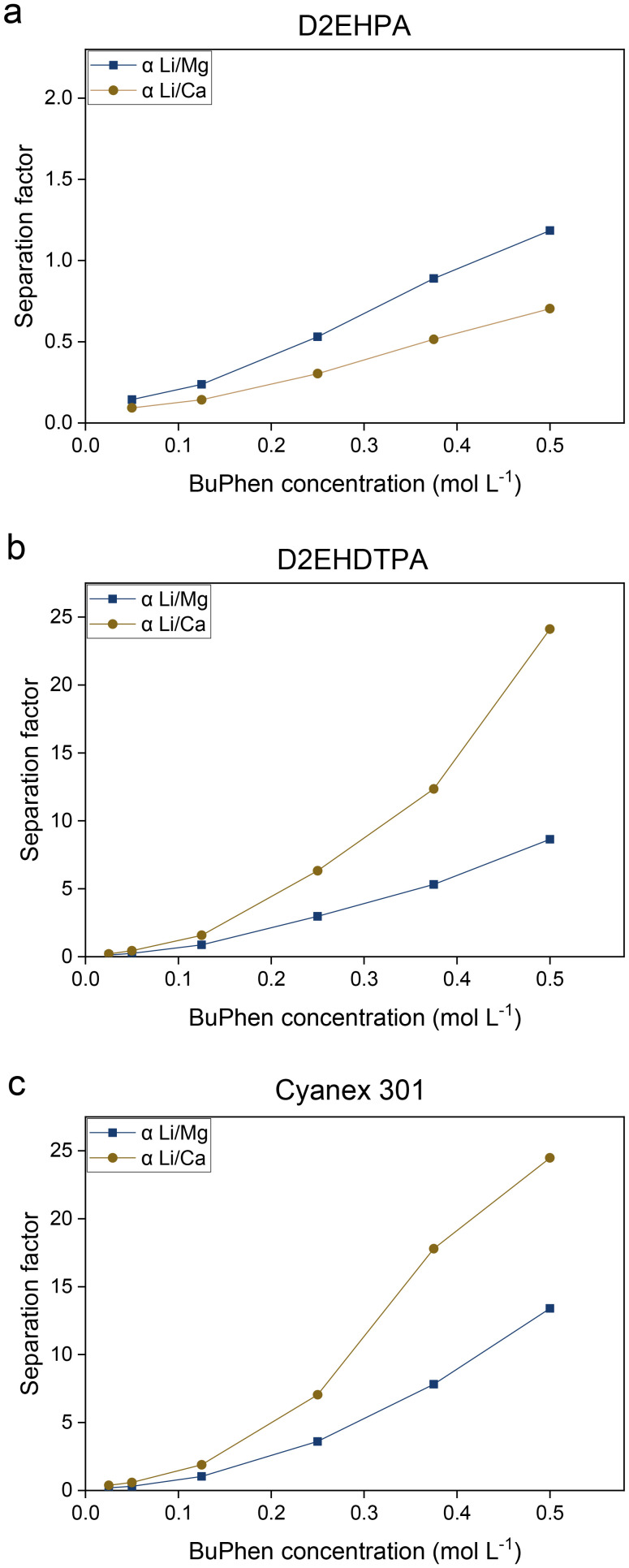
Separation factors *α*_Li/Mg_ and *α*_Li/Ca_ as a function of the BuPhen concentration for various saponified extractants (0.05 mol L^−1^): di(2-ethylhexyl)phosphoric acid (D2EHPA, a), di(2-ethylhexyl)dithiophosphoric acid (D2EHDTPA, b) and bis(2,4,4-trimethylpentyl)dithiophosphinic acid (Cyanex 301, c). Extractants were diluted in *n*-dodecane with 10 vol% 1-octanol and 2-octanol. The aqueous phase contained 0.05 mol L^−1^ each of LiCl, KCl, RbCl, MgCl_2_ and CaCl_2_. Phase volume ratio: 1 : 1, equilibrium temperature 25 °C.

The extractant used in the LiSX™ process is Cyanex® 936P, developed by Solvay (currently Syensqo). The composition of this extractant has not been disclosed, but it is described as being phosphorus-based. The extraction behavior of this extractant is consistent with that of β-diketone (Fig. 1, 1)/phosphine oxide (Fig. 1, 2) mixtures described in the older literature.^17–21^ These extractants are also characterized by a selectivity in order of the charge density of the cations, *i.e.* Mg^2+^ > Ca^2+^ > Li^+^ > Na^+^ ≈ K^+^. Extraction is suppressed with increasing acidity, as extraction occurs *via* a cation exchange mechanism, in which protons are exchanged for metal ions. The synergist employed in the literature is usually a trialkyl phosphine oxide (*e.g.* Cyanex® 923), as these ligands greatly improve the selectivity of the extraction system for lithium. Nevertheless, organophosphates (Fig. 1, 4), alcohols, amides and a number of other ligands also exhibit a certain degree of synergism, and solvents without synergists have also been used.^19,20,22^ Ishimori *et al.* showed that the use of sterically hindered, bidentate ligands based on 1,10-phenanthroline (Fig. 1, 3) improves the selectivity of lithium over other alkali metals in comparison to phosphine oxides.^23–25^ These ligands are known to selectively coordinate to lithium ions over other alkali metal ions.^26^ This property has been attributed to the propensity of the lithium ion to adopt a tetrahedral coordination sphere with four donor atoms. Other alkali metal ions, as well as alkaline earth ions, exhibit a strong preference for octahedral coordination with six donor atoms, a geometry that cannot be attained with more sterically hindered ligands.^24,26^

**Fig. 5:**
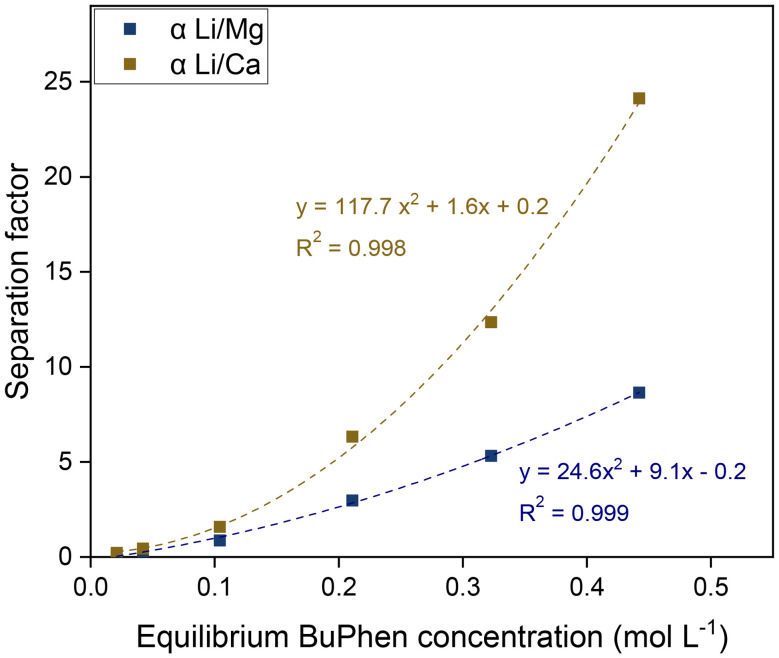
Separation factors *α*_Li/Mg_ and *α*_Li/Ca_ as a function of the equilibrium BuPhen concentration for 0.050 mol L^−1^ saponified D2EHDTPA diluted in *n*-dodecane with 10 vol% 1-octanol and 2-octanol. The aqueous phase contained 0.050 mol L^−1^ each of LiCl, KCl, RbCl, MgCl_2_ and CaCl_2_. Phase volume ratio: 1 : 1, equilibrium temperature: 25 °C.

Phosphoric acid extractants, such as bis(2-ethylhexyl)phosphoric acid (D2EHPA) and mono(2-ethylhexyl)phosphoric acid (Fig. 1, 5), are capable of extracting lithium at relatively high pH, in synergism with tri-*n*-butyl phosphate.^27^ The mechanism is similar to the β-diketone/phosphine oxide system in that the acidic component functions as a cation exchanger, and the neutral component serves to saturate the coordination sphere. While this system extracts lithium efficiently, it also preferentially extracts magnesium and the selectivity over other alkali metal ions is poor.

A number of solvent extraction systems have been investigated that can selectively extract lithium over alkaline earth metals, but these have serious drawbacks that preclude their implementation in industrial settings. Lithium can be selectively extracted from magnesium-rich matrices by the tri-*n*-butyl phosphate (TBP, Fig. 1, 4)/iron(iii) chloride system.^28–34^ The organic phase contains the TBP – sodium tetrachloroferrate(iii) complex, which readily exchanges sodium for lithium, but not magnesium. This lithium-bearing complex can be decomposed by contacting the organic phase with hydrochloric acid solution, and subsequently deprotonating the resulting HFeCl_4_ to regenerate the solvent. This process has several drawbacks: the distribution ratios for lithium are relatively low, large amounts of sodium are co-extracted and cannot readily be removed by scrubbing of the organic phase, and very high organic-to-aqueous phase ratios (around 20 : 1 to 40 : 1) and chloride concentrations are required during scrubbing and stripping to prevent iron losses.^31^ If 2-ethylhexylphosphonic acid mono(2-ethylhexyl) ester (P507) is added to the organic phase, stripping can be accomplished using water, albeit in similarly high phase ratios.^35,36^ Su *et al.* reported the gradual loss of iron under these conditions.^35^ Yu *et al.* investigated a 5-stage continuous extraction process based on the tris(isobutyl)phosphate/FeCl_3_/P507 system, and found that loss of iron was significant when using a more practical phase ratio of 2 : 1.^37^

The tetrachloroferrate(iii) anion mainly serves as a non-coordinating counterion in the TBP/FeCl_3_ system, and accordingly a number of conceptually similar systems have been identified that combine a weakly coordinating cation exchanger with TBP or another synergist.^34^ The synergist binds lithium to confer the desired selectivity to the system, but the cation exchanger is required to drive the extraction itself, as the synergists on their own are insufficiently strong extractants to sequester the strongly solvated alkali metal ions from the aqueous phase. Among the TBP-based extraction systems are those with the ionic liquid 1-ethyl-3-methylimidazolium bis(trifluoromethylsulfonyl)imide and sodium tetraphenylborate as cation exchangers.^38–40^ The former exchanges organic cations for lithium and is hence industrially unviable, while both suffer from high aqueous solubility of the cation exchanger.

**Fig. 6:**
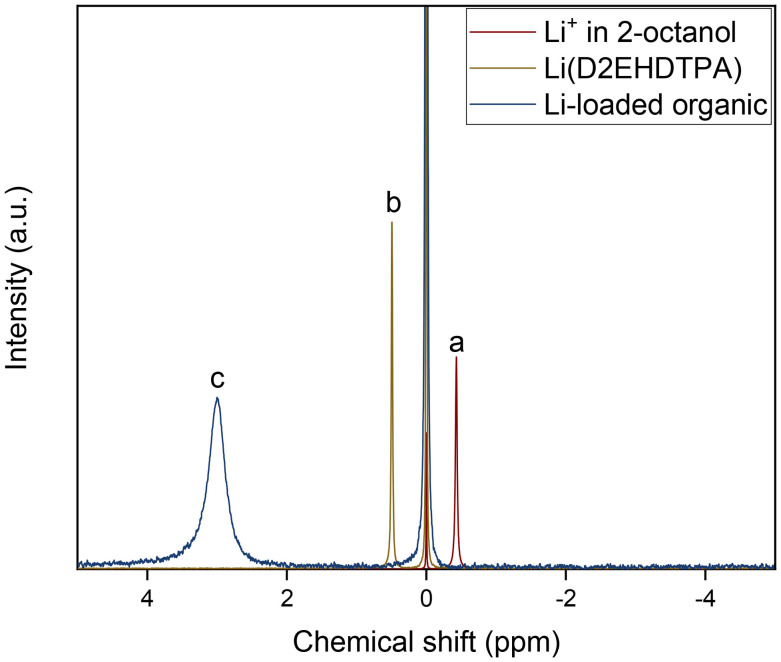
^7^Li NMR spectra of (a) 2-octanol saturated with lithium bis(trifluoromethylsulfonyl)imide, (b) 0.050 mol L^−1^ of D2EHDTPA in *n*-dodecane, loaded with Li^+^, and (c) 0.050 mol L^−1^ of Li(D2EHDTPA) and 0.100 mol L^−1^ of BuPhen in *n*-dodecane with 2.5 vol% 1-octanol and 20 vol% 2-octanol, loaded with Li^+^. Spectra are referenced to 1.0 mol L^−1^ of LiCl in D_2_O (0.00 ppm). Spectra have been scaled for clarity.

Very high selectivity for lithium over other metal ions can be achieved using extractants based on crown ethers and calix[4]pyrroles, but these compounds are difficult to prepare and very expensive, rendering them unsuitable for industrial applications.^41–43^ The highest selectivity for lithium was obtained using the small 12-crown-4 (Fig. 1, 6) macrocycle.^43^

In this work, a new solvent is presented for lithium extraction, composed of a water-insoluble cation exchanger, bis(2-ethylhexyl)dithiophosphoric acid (D2EHDTPA) (Fig. 2); a lithium-selective synergist, 2,9-dibutyl-1,10-phenanthroline (BuPhen) (Fig. 3); two modifiers, 1-octanol and 2-octanol, to prevent third-phase and gel formation; and an aliphatic diluent, *n*-dodecane.

D2EHDTPA itself is somewhat selective for divalent metal cations over lithium, albeit weakly so. The inclusion of BuPhen shifts the cation exchange equilibrium towards the extraction of lithium by forming a stable, tetrahedral complex. This ligand has been described as a lithium-selective complexing agent in the context of lithium-selective electrode design, and was found to possess a higher selectivity over other alkali and alkaline earth ions than other 1,10-phenanthroline derivatives.^26^

## Results and discussion

### Synthesis of extractants

The synthesis of extractants is discussed in detail in the ESI (page S4–S6†). Bis(2-ethylhexyl)dithiophosphoric acid (D2EHDTPA) was conveniently prepared from P_4_S_10_ and 2-ethylhexanol, and purified by filtration and washing of the reaction mixture.^44^ This afforded the product in a yield of 88%. 2,9-Dibutyl-1,10-phenanthroline was prepared by addition of *n*-butyllithium to 1,10-phenanthroline, followed by oxidative rearomatization using MnO_2_.^45^ The crude product was purified by recrystallization from heptane. Crystals of BuPhen were obtained in a yield of 64%.

### Solvent extraction

Solvent extraction experiments were carried out in 4 mL vials. The concentration of D2EHDTPA in the organic phase was chosen to be stoichiometric with the amount of lithium in the aqueous phase, in order to improve separation through saturation effects. The equilibrium temperature was 25 °C and the phase volume ratio was 1 : 1, unless stated otherwise.

The distribution ratio (*D*) is used as a metric for the degree of extraction (eqn (1)), defined as the ratio of the equilibrium organic concentration of metal M ([M]_org_) to the equilibrium aqueous concentration of the same metal ([M]_aq_):1
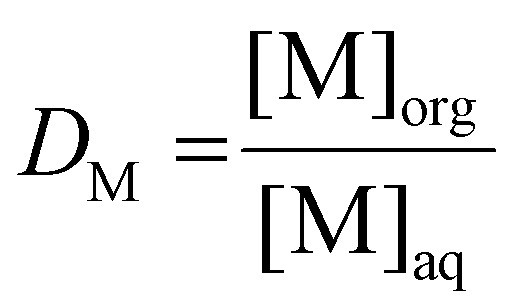


As far more accurate and precise analyses can be obtained for aqueous solutions than for organic solutions, the distribution ratio is inferred by measuring the aqueous phase before and after extraction. Assuming no significant changes in phase volume ratio occur, the distribution ratio can be calculated using eqn (2):2
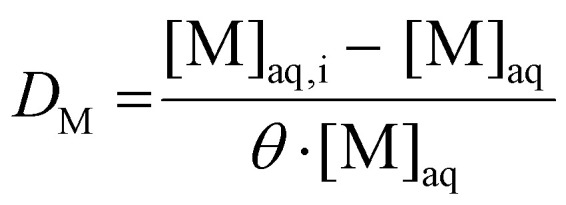
wherein [M]_aq,i_ denotes the initial concentration of metal M in the feed solution, and *θ* is the phase volume ratio, defined as the ratio of the volume of the organic phase to that of the aqueous phase.

Similarly, the percentage extraction (%*E*) can be calculated by analysis of the aqueous phase before and after extraction (eqn (3)), assuming no changes in phase volume ratio occur:3
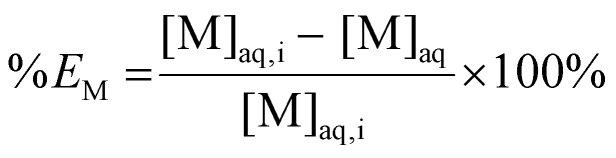


An analogous calculation can also be made for the transfer of metal ions from a loaded organic solution to a barren aqueous stripping liquor, to yield the percentage stripping (%*S*). The concentration of the metal in the organic phase is then inferred from the concentration in the aqueous phase before and after loading of the solvent.

The selectivity of the process is quantified in terms of the separation factor (*α*), defined according to eqn (4):4
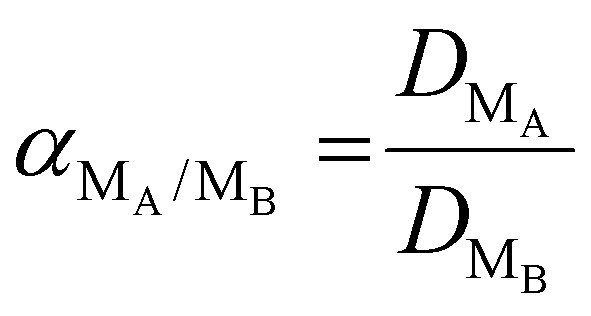


By convention, the separation factor is defined to be greater than unity, *i.e.* having the most strongly extracted metal in the numerator of eqn (4).

### Selection of the cation exchanger and concentration

The lithium-selectivity of the synergistic extraction system is contingent on the absence of specific interactions between the cation exchanger and any of the metal cations, in order to allow free exchange of cations and complexation of lithium by the synergist. Three cation exchangers were evaluated for this property: bis(2-ethylhexyl)phosphoric acid (D2EHPA), bis(2-ethylhexyl)dithiophosphoric acid (D2EHDTPA) and bis(2,4,4-trimethylpentyl)dithiophosphinic acid (formerly sold as Cyanex® 301, currently commercially available as Mextral® 301). D2EHPA is an attractive candidate due to its low price and widespread use in solvent extraction. On the other hand, thiophosphorus acids are expected to perform well in light of the HSAB principle (hard and soft acids and bases), which predicts weak interactions between the soft sulfur donor atoms of the extractant and the hard alkali and alkaline earth cations. Indeed, Cyanex 301 is known to be a poor extractant for Ca^2+^ and Mg^2+^.^46^ The performance of these extractants as cation exchangers was assessed by equilibrating an aqueous solution containing 0.050 mol L^−1^ each of LiCl, KCl, RbCl, MgCl_2_ and CaCl_2_ with solvents containing the extractants in their sodium-loaded form. The organic phase comprised a 0.050 mol L^−1^ solution of the saponified extractant in *n*-dodecane with varying concentrations of BuPhen, ranging from 0.05 to 0.5 mol L^−1^. Both 1-octanol and 2-octanol were added as modifiers (10 vol%). The concentration of the cation exchanger was deliberately chosen to be stoichiometric with respect to the amount of lithium to be extracted. This improved the selectivity due to saturation effects: as the cation exchanger will always retain the number of cations needed to balance its charge, using a superstoichiometric amount will inevitably lead to coextraction of undesirable ions, such as Ca^2+^ or Mg^2+^.

The results of this assay, shown in Fig. 4, demonstrate that thiophosphorus acids are indeed better suited for the synergistic extraction system than D2EHPA. While the separation factors *α*_Li/Mg_ and *α*_Li/Ca_ improve with increasing concentration of BuPhen for all cation exchangers, selectivity of lithium over calcium is only achieved using thiophosphorus-based cation exchangers, as evidenced by *α*_Li/Ca_ being greater than unity. A very slight selectivity for lithium over magnesium was achieved using 0.5 mol L^−1^ of BuPhen and D2EHPA, but the D2EHPA system remained selective for alkaline earth ions over lithium at all other BuPhen concentrations. D2EHPA is known to be a relatively strong extractant for alkaline earth metal ions, in particular Ca^2+^, explaining the findings presented in Fig. 4a.^46^ While separation factors are slightly more favorable for Cyanex 301 (Fig. 4c) than for D2EHDTPA (Fig. 4b), the latter was nevertheless chosen for further study. The lower acidity of Cyanex 301 causes extraction of protons from the feed. This leads to a loss of capacity of the organic phase, as well as potential precipitation of magnesium at higher concentrations.

### Optimization of modifier concentration

Two modifiers were used: 2-octanol and 1-octanol. 2-Octanol was found to be effective at preventing gel formation in systems containing only saponified D2EHDTPA in *n*-dodecane. Upon addition of higher concentrations of BuPhen, third-phase formation can occur unless a sufficient amount of 1-octanol is added as well. Unfortunately, these modifiers were found to adversely affect the separation factors of the extraction system. Therefore, the minimal required concentrations of either modifier were determined at a BuPhen concentration of 0.25 mol L^−1^ and a saponified D2EHDTPA concentration of 0.050 mol L^−1^. The aqueous phase consisted of a 0.050 mol L^−1^ solution of LiCl, KCl, RbCl, MgCl_2_ and CaCl_2_. Three concentrations of 2-octanol were tested (0, 10 and 20 vol%). 1-Octanol was added until the point at which the third phase resolved. This occurred at 10 vol% 1-octanol for 0 vol% 2-octanol, at 5 vol% 1-octanol for 10 vol% 2-octanol, and at 2.5 vol% 1-octanol for 20 vol% 2-octanol. The associated separation factors *α*_Li/Mg_ were 8.2, 11.6 and 11.1, respectively. The latter system was found to be particularly robust: addition of a minimal amount of 1-octanol resulted in the immediate homogenization of the organic phase. On the basis of this observation and the measured separation factors, the latter condition (2.5 vol% 1-octanol and 20 vol% 2-octanol) condition was selected for further study. The use of an aromatic diluent (*p*-cymene) as opposed to *n*-dodecane did not eliminate the need for either 1-octanol or 2-octanol and, hence, was not further investigated.

### Study of the extraction mechanism

While it is evident from the extraction results in Fig. 4b that selectivity is conferred by BuPhen and that D2EHDTPA behaves as a weakly coordinating ion exchanger, further study was carried out to elucidate the exact mechanism of extraction and the speciation of the extracted metals. The first clue is given by the apparent quadratic trend in the separation factors as a function of the BuPhen concentration. This points to a 1 : 2 stoichiometry between Li^+^ and BuPhen. Assuming this mechanism and taking into account the fraction of free BuPhen that would be removed from the solution by complexation, this trend can be very accurately fitted, with a coefficient of determination (*R*^2^) of 0.999 for *α*_Li/Mg_ and 0.998 for *α*_Li/Ca_ (Fig. 5).

Respecting the 1 : 2 stoichiometry of Li^+^ and BuPhen and considering the function of D2EHDTPA as an ion exchanger, the following reactions are postulated to describe extraction equilibria:5
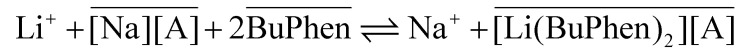
with equilibrium constant *K*_Li_, and:6
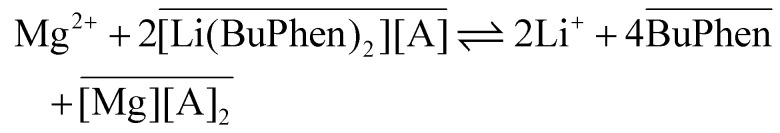
with equilibrium constant *K*_MgLi_. In eqn (5) and (6), overbars denote species in the organic phase, while A^−^ represents the D2EHDTPA anion.

From the formulae for the equilibrium constants:7
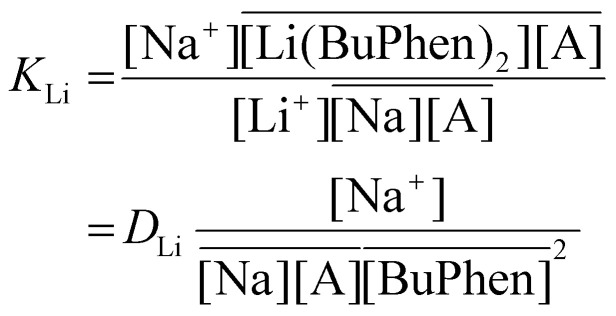
and8
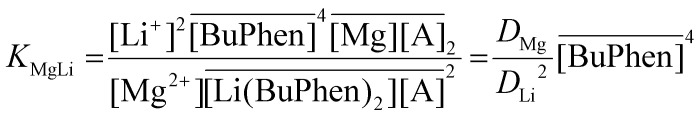
it follows that:9
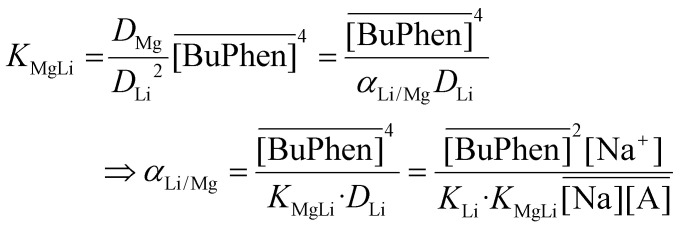


Eqn (9) explains both the quadratic relationship between the separation factor and the equilibrium BuPhen concentration, as well as the fact that the relationship is not purely of second order. The distribution of sodium will change due to the shifting of the extraction equilibria, and this nonquadratic influence on the value of *α* will lead to the emergence of lower order terms in the fit (even if this influence is not strictly described by a first-order polynomial). In subsequent paragraphs, eqn (9) is shown to be key in understanding the response of the extraction equilibrium to other changes in the composition of the feed and organic phase, as well.

X-nucleus NMR was also used to probe interactions in the loaded organic phase and to support the hypothetical extraction mechanism. The chemical environment of extracted Li^+^ ions was investigated using ^7^Li NMR spectroscopy (Fig. 6). The spectrum of the organic phase (0.050 mol L^−1^ of D2EHDTPA, 0.100 mol L^−1^ of BuPhen, 2.5 vol% 1-octanol and 20 vol% 2-octanol in *n*-dodecane) loaded with lithium was compared to that of 1.00 mol L^−1^ of LiCl in D_2_O (used as reference), 0.050 mol L^−1^ of D2EHDTPA saponified with LiOH in *n*-dodecane, and saturated lithium bis(trifluoromethylsulfonyl)imide in 2-octanol. The latter three spectra serve as representatives for Li^+^ solvated strictly by D_2_O, D2EHDTPA and by 2-octanol, respectively. It is clear that ^7^Li resonates several ppm further downfield in the organic phase than would be expected if Li^+^ were solvated by D2EHDTPA or an alcohol, or if Li^+^ were simply hydrated. This downfield shift can be explained by coordination of the aromatic BuPhen ligand: this coordination places the lithium cation in the region of magnetic anisotropy induced by aromatic ring currents.

Similarly, ^13^C NMR spectra indicate coordination of Li^+^ to BuPhen (Fig. S1, ESI†). The spectrum of the organic phase saponified with Li^+^ and equilibrated with water, was compared to that of the organic loaded with Mg^2+^ and equilibrated with water, and the organic phase without D2EHDTPA or metal ions_._ In the Li-loaded organic phase, most ^13^C nuclei in the BuPhen ring system show a downfield shift between 0.3 and 1.4 ppm, which is attributable to the electron-withdrawing effect of the positively charged Li^+^ cation. The NMR spectrum of the organic phase loaded with Mg^2+^ largely coincides with that of the organic phase without D2EHDTPA or metal ions, indicating negligible interaction between Mg^2+^ and BuPhen. Interestingly, the strongest shift (approx. 1.4 ppm) and broadening is observed for the carbon-13 nuclei in *para* to the nitrogen atom. Pazderski *et al.* observed a similar trend in the ^13^C chemical shifts of a gold(iii) phenanthroline complex, along with a slight upfield shift for one of the *ortho* carbon-13 nuclei.^47^ An upfield shift (approx. 0.6 ppm) is also observed for the benzo *ortho* carbon in BuPhen upon loading with lithium. In the spectra of the organic phase loaded with Mg^2+^, the *para* carbon nucleus shifts approximately 0.05 ppm downfield with respect to the unloaded organic phase. This shift is a factor 28 smaller than that observed for the organic phase loaded with Li^+^, irrespective of the higher charge of the Mg^2+^ cation.

Finally, ^31^P NMR was used to gauge the strength of the interactions between D2EHDTPA and the extracted metal ions (Fig. S2, ESI†). In spite of the fact that D2EHDTPA is present in stoichiometric quantities with respect to the extracted metal ions, the difference in ^31^P signals of the dithiophosphate group in the Li^+^ loaded *vs.* the Mg^2+^ loaded organic phase is no more than 0.07 ppm. D2EHDTPA thus appears to interact only weakly with the extracted metal ions, as is expected in light of the HSAB principle. Most likely, the D2EHDTPA anions are present in the second coordination sphere of the extracted metal ions.

### Variation of the BuPhen and D2EHDTPA concentrations

**Fig. 7:**
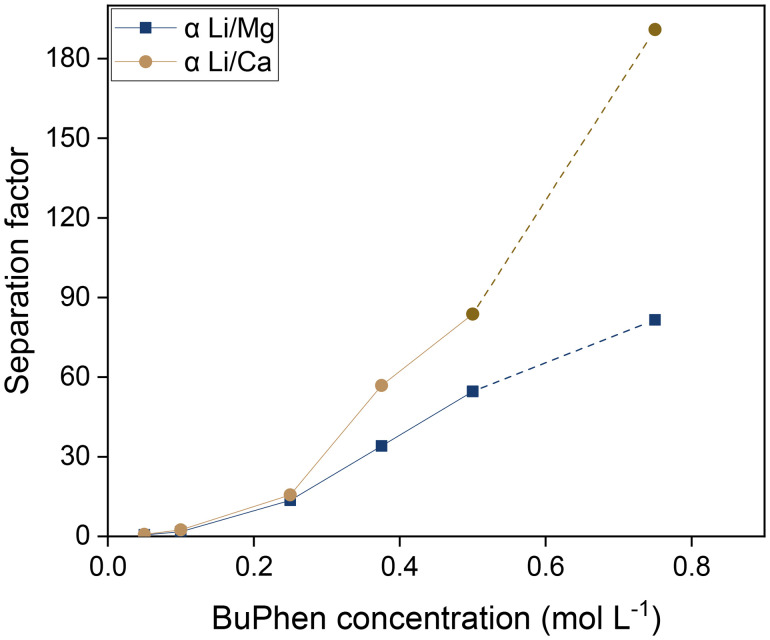
Influence of the BuPhen concentration on the separation factors. Organic phase: 0.05 mol L^−1^ saponified D2EHDTPA, 2.5 vol% 1-octanol (5 vol% for 0.750 mol L^−1^ BuPhen), 20 vol% 2-octanol, diluted in *n*-dodecane. Aqueous phase: 0.050 mol L^−1^ each of LiCl, KCl, RbCl, MgCl_2_ and CaCl_2_. Phase volume ratio: 1 : 1, equilibrium temperature: 25 °C.

The dependence of the separation factors on the BuPhen concentration was investigated under optimized conditions, *i.e.* 2.5 vol% 1-octanol and 20 vol% 2-octanol (Fig. 7). The BuPhen concentration was varied between 0.050 mol L^−1^ and 0.750 mol L^−1^. The concentration of 1-octanol was increased to 5 vol% for the sample with a BuPhen concentration of 0.750 mol L^−1^. Note that this concentration does not correspond to the solubility limit of BuPhen and that higher concentrations are possible. These concentrations may, however, require a further increase in the concentration of 1-octanol in order to prevent third phase formation. The concentration of saponified D2EHDTPA was fixed at 0.050 mol L^−1^. The aqueous phase comprised LiCl, KCl, RbCl, MgCl_2_ and CaCl_2_, each in a concentration of 0.05 mol L^−1^.

The results in Fig. 7 show a progressive increase of the separation factors with increasing BuPhen concentrations, demonstrating that this component is pivotal in obtaining the desired selectivity for lithium over alkaline earth metals. Furthermore, the high BuPhen concentration of 0.750 mol L^−1^ of BuPhen results in the highest separation factors, in spite of any detrimental effect caused by the increased concentration of 1-octanol. At this point, *α*_Li/Mg_ = 82 and *α*_Li/Ca_ = 191. The following percentages extraction were obtained: %*E*_Li_ = 81%, %*E*_Mg_ = 5%, %*E*_Ca_ = 2%, %*E*_Na_ = 4%, %*E*_K_ = 2% and %*E*_Rb_ = 2%.

**Fig. 8:**
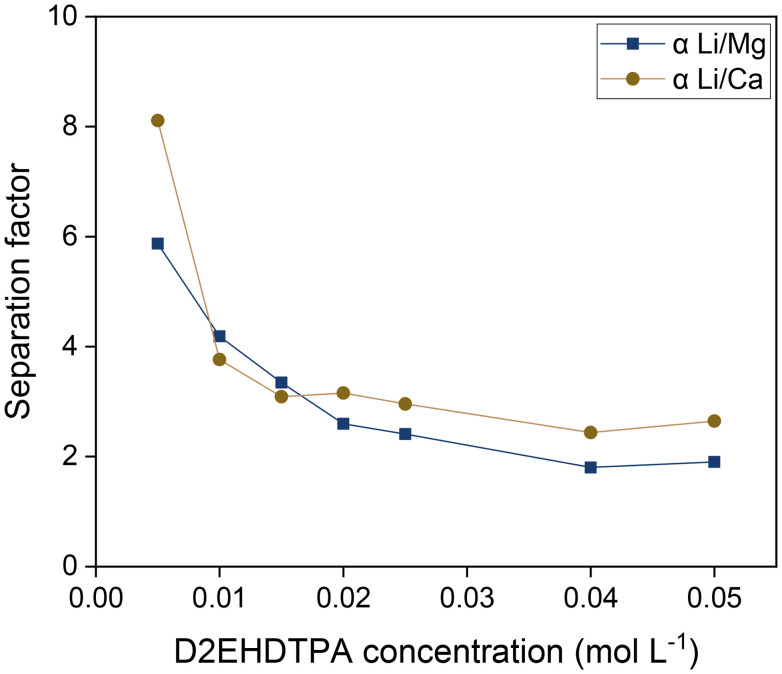
Influence of the saponified D2EHDTPA concentration on the separation factors. Organic phase: 0.125 mol L^−1^ of BuPhen, 2.5 vol% 1-octanol, 20 vol% 2-octanol, diluted in *n*-dodecane. Aqueous phase: 0.050 mol L^−1^ each of MgCl_2_ and CaCl_2_, 50 g L^−1^ of NaCl, and equistoichiometric LiCl with respect to D2EHDTPA. Phase volume ratio: 1 : 1, equilibrium temperature: 25 °C.

Similarly, a variation of the D2EHDTPA concentration was also conducted (Fig. 8). The aqueous LiCl concentration was commensurately varied to exploit saturation effects at every D2EHDTPA concentration. The organic phase was composed of varying concentrations of saponified D2EHDTPA, 0.125 mol L^−1^ of BuPhen, 2.5 vol% 1-octanol and 20 vol% 2-octanol. A low concentration of BuPhen was used, as trends in separation factors can be more accurately determined at lower values of *α*. The aqueous phase contained varying concentrations of LiCl, and 0.050 mol L^−1^ each of MgCl_2_ and CaCl_2_. In addition, a constant NaCl concentration of 50 g L^−1^ was introduced in order to facilitate interpretation of the results. In accordance with eqn (9), selectivity for lithium improves with decreasing D2EHDTPA concentrations. As the aqueous concentration of sodium was far greater than the amount of sodium extracted to the organic phase, the sodium concentration can be viewed as *de facto* constant. Under this constraint, the separation factors *α*_Li/Mg_ and *α*_Li/Ca_ are determined entirely by the equilibrium BuPhen concentration and the extracted sodium concentration. The former can be calculated from the loading of lithium and rises at low extracted lithium concentrations, resulting in a further increase of the separation factors. However, eqn (9) does not imply a strict inverse relationship between the analytical D2EHDTPA concentration and the product 
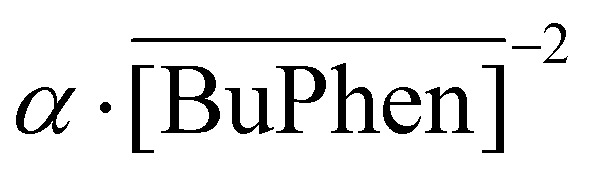
, as the fraction of D2EHDTPA loaded with sodium is itself a function of the feed and organic phase composition.

### Variation of the NaCl concentration

**Fig. 9:**
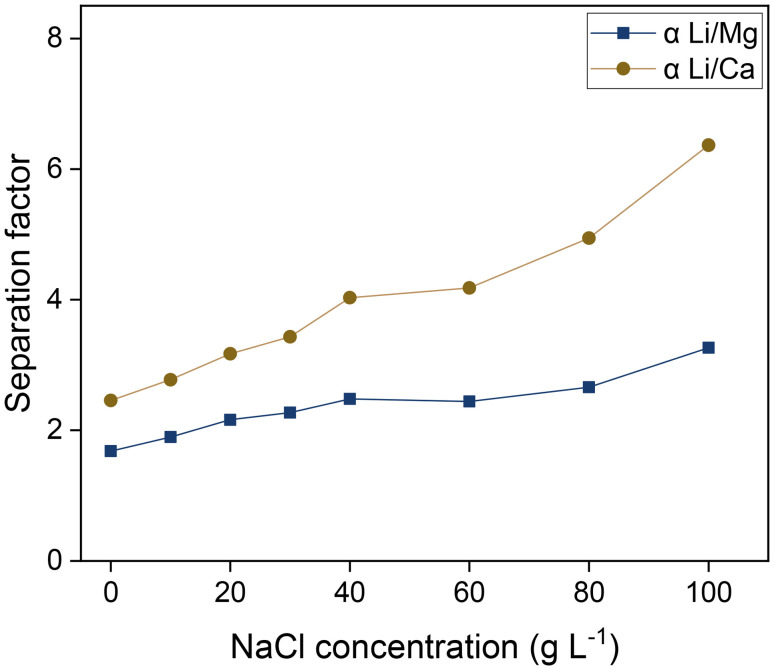
Influence of the NaCl concentration on the separation factors. Organic phase: 0.050 mol L^−1^ of saponified D2EHDTPA 0.125 mol L^−1^ of BuPhen, 2.5 vol% 1-octanol, 20 vol% 2-octanol, diluted in *n*-dodecane. Aqueous phase: 0.050 mol L^−1^ each of LiCl, MgCl_2_ and CaCl_2_, and a variable NaCl concentration. Phase volume ratio: 1 : 1, equilibrium temperature: 25 °C.

According to eqn (9), the concentration of sodium in the feed also strongly affects the separation of lithium and alkaline earth metals. An investigation of this effect is paramount considering the high sodium concentrations present in natural brines. To this end, an experiment was conducted in which the NaCl concentration was varied up to 100 g L^−1^, corresponding to 39.3 g L^−1^ of sodium (Fig. 9). The organic phase comprised 0.050 mol L^−1^ saponified D2EHDTPA, 0.125 mol L^−1^ of BuPhen 2.5 vol% 1-octanol and 20 vol% 2-octanol. The aqueous phase contained 0.050 mol L^−1^ of LiCl, MgCl_2_ and CaCl_2_. This experiment was performed in duplicate, and the resulting average separation factors are shown. As expected, separation factors increase as the concentration of sodium increases.

Intuitively, the dependence of the separation factors on the concentrations of D2EHDTPA and sodium can be understood in terms of the stoichiometry of the ion-exchange equilibria. Extraction of a lithium ion requires the expulsion of only one sodium ion, while the extraction of a divalent ion requires the expulsion of two sodium ions. Hence, the distribution ratios of divalent ions show a quadratic dependence on the sodium concentration, while that of lithium ions exhibits a linear dependence. As a result, the extraction of divalent ions becomes disproportionally more unfavorable as the sodium concentration increases. Similarly, two D2EHDTPA counterions are required for the extraction of an alkaline earth ion, while only one is required per lithium ion, leading to improved selectivity at low D2EHDTPA concentrations.

### Influence of the equilibrium temperature

**Fig. 10:**
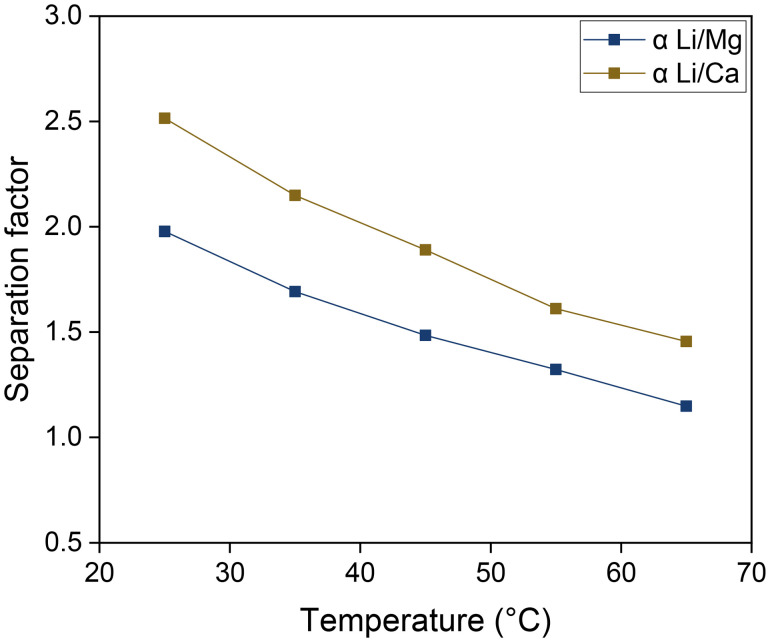
Influence of the equilibrium temperature on the separation factors. Organic phase: 0.050 mol L^−1^ of D2EHDTPA saponified with LiOH, 0.125 mol L^−1^ of BuPhen, 2.5 vol% 1-octanol, 20 vol% 2-octanol, diluted in *n*-dodecane. Aqueous phase: 0.050 mol L^−1^ each of MgCl_2_ and CaCl_2_. Phase volume ratio: 1 : 1.

The temperature dependence of the selectivity is an important aspect to consider if the DLE process is to be applied to geothermal brines, which are extracted at elevated temperatures. This was studied by equilibrating the two phases at a range of temperatures between 25 and 65 °C (Fig. 10). The organic phase was composed of 0.050 mol L^−1^ of D2EHDTPA saponified with LiOH, 0.125 mol L^−1^ of BuPhen, 2.5 vol% 1-octanol and 20 vol% 2-octanol. The aqueous phase comprised 0.050 mol L^−1^ of MgCl_2_ and CaCl_2_. The separation factors *α*_Li/Mg_ and *α*_Li/Ca_ were found to decrease with increasing temperatures over the tested temperature range, indicating that substitution of magnesium or calcium by lithium is an exothermic process. Thus, cooling of geothermal brines to ambient temperature would benefit the solvent extraction process.

**Fig. 11:**
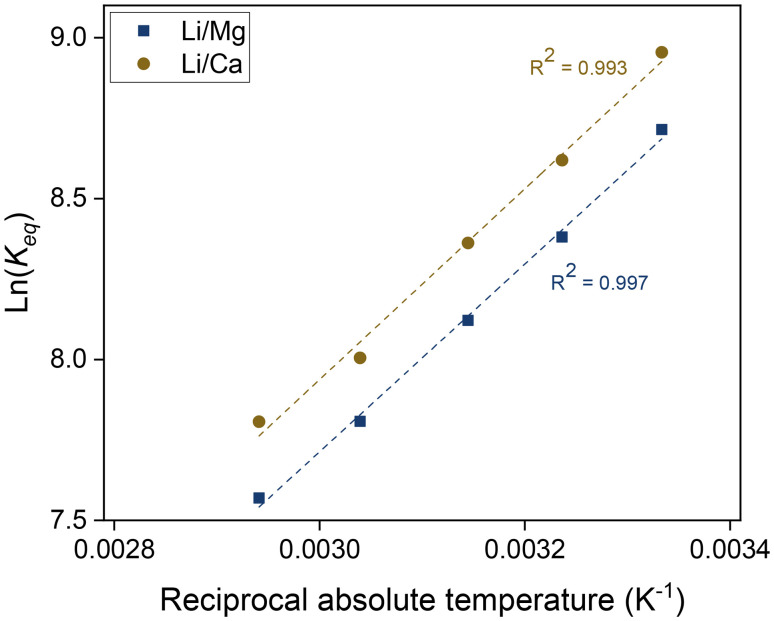
Van't Hoff plot of the ion-exchange reactions between lithium and magnesium and between lithium and calcium. Organic phase: 0.050 mol L^−1^ of D2EHDTPA saponified with LiOH, 0.125 mol L^−1^ of BuPhen, 2.5 vol% 1-octanol, 20 vol% 2-octanol, diluted in *n*-dodecane. Aqueous phase: 0.050 mol L^−1^ each of MgCl_2_ and CaCl_2_. Phase volume ratio: 1 : 1.

The temperature dependence of the separation factors can also be used to determine the thermodynamic parameters of the Li/Mg exchange equilibrium given by eqn (6). First, the equilibrium constants of the exchange were obtained from the separation factors by means of eqn (8). The equilibrium constants for the Li/Ca exchange were derived analogously. A Van't Hoff plot of these data is shown in Fig. 11, alongside a linear regression. Details of the regression are given in the ESI (Table S1†). According to the Van't Hoff method, which is derived from the Gibbs–Helmholtz equation:10
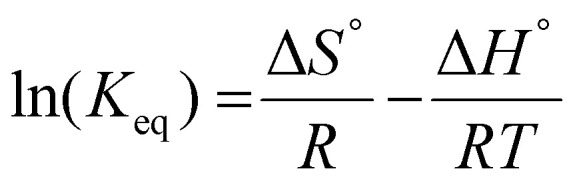
where *T* represents the absolute temperature (in kelvin). This implies that the enthalpy of exchange (Δ*H*°) can be obtained by multiplying the slope of the regression by the universal gas constant (*R*) and −1. This yields 
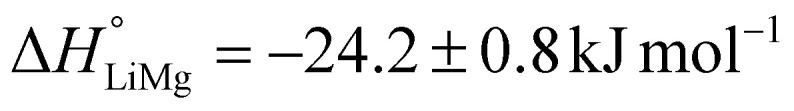
 and 
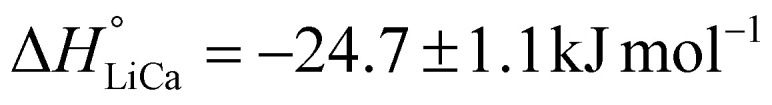
. The entropy of reaction (Δ*S*°) can theoretically be calculated by multiplying the intercept of the curve by *R*. However, the necessity of extrapolating the regression to *T*^−1^ = 0, far outside the range of available data, results in a very poor estimate of the intercept. The following values are obtained: 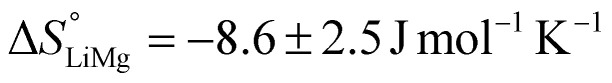 and 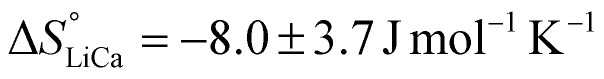, both with high relative standard errors. The need for extrapolation of the regression can be circumvented by using the Gibbs–Helmholtz equation directly to calculate the Δ*S*° from Δ*H*° and the standard Gibbs free energy of reaction Δ*G*° (eqn (11)):

11 Δ*G*° = *RT* ln(*K*_eq_) = Δ*H*° − *T*Δ*S*°

Repeating this calculation for every value of *T* and averaging yields 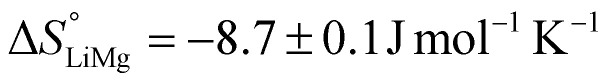 and 
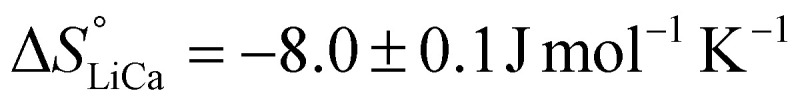
. These values accord well with those obtained by the Van't Hoff method and demonstrate that the entropy change upon substitution of magnesium by lithium is significantly more unfavorable than that associated with the displacement of calcium (difference 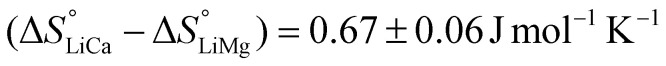, 95%CI [0.49, 0.85], *p*-value = 0.0005 in two-tailed, paired *t*-test: see ESI†). The unfavorable entropy of the substitution of Mg^2+^ by Li^+^ may result from the strong hydration of the Mg^2+^ ion, and hence strong ordering of the water molecules in the aqueous phase. Conversely, the values obtained for Δ*H*° using the Van't Hoff method do not differ significantly from each other, as the assay lacks the necessary statistical power to determine which of these values is more favorable.

### Stripping of the loaded metal ions

**Fig. 12:**
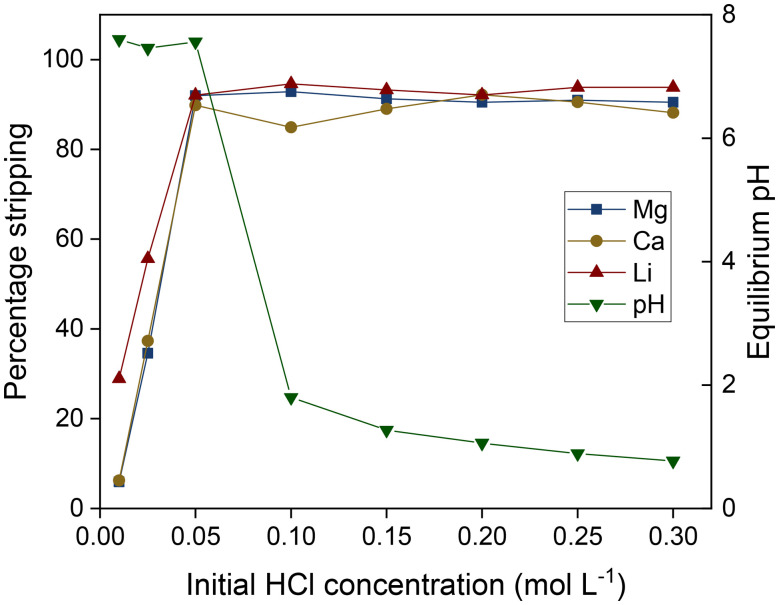
Stripping of lithium, magnesium and calcium from a loaded organic phase consisting of saponified D2EHDTPA (0.050 mol L^−1^), BuPhen (0.250 mol L^−1^), 2,5 vol% 1-octanol and 20 vol% 2-octanol in *n*-dodecane, loaded from a solution of LiCl, MgCl_2_ and CaCl_2_ (0.050 mol L^−1^ each). Phase volume ratio: 1 : 1, equilibrium temperature: 25 °C.

As the extraction of ions is contingent on the availability of deprotonated D2EHDTPA molecules in the organic phase, stripping can be achieved using acidic aqueous solutions. This method also allows the counterion of the recovered lithium salts to be selected by proper choice of the acid used for stripping. Stripping was tested on a loaded organic phase, prepared by contacting 8 mL of a solution of saponified D2EHDTPA (0.050 mol L^−1^) and BuPhen (0.250 mol L^−1^) in *n*-dodecane (modified with 2.5 vol% 1-octanol and 20 vol% 2-octanol) with an aqueous salt solution containing 0.050 mol L^−1^ each of LiCl, MgCl_2_ and CaCl_2_. Aliquots (400 μL) of this solution were then stripped by contacting with equal volumes of aqueous HCl solutions of varying concentration. Because of the equal volumes of the phases, an aqueous HCl concentration of 0.050 mol L^−1^ corresponds to a stoichiometric amount of acid in the aqueous phase for full protonation of D2EHDTPA. As shown in Fig. 12, this was sufficient to reach the maximum value of %*S*. The values for %*S* appears to plateau at about 94% for lithium and 91% for the alkaline earth metals. This deviation from 100% is likely due to a cumulation of analytical errors and phase volume changes during the extraction and stripping steps. Sub-stoichiometric amounts of acid did not result in selective scrubbing of calcium and magnesium.

The pH of the stripping solution remains between 7 and 8 until a stoichiometric amount of HCl is added, beyond which point the pH drops sharply to below 2. These low pH values were associated with a striking color change to a reddish hue in both phases. These observations indicate that selective protonation of D2EHDTPA occurs first, at a pH of about 7.5. Further addition of acid will result in the protonation of BuPhen at pH values below 2, accompanied by the loss of BuPhen to the aqueous phase. The components of the organic phase act as buffers, resulting in the aqueous pH being relatively stable until the buffer capacity of D2EHDTPA is exceeded (*i.e.* full protonation of D2EHDTPA). In order to prevent losses of BuPhen, stripping should only be performed with stoichiometric amounts of acid, as this is already sufficient for full recovery of all extracted metal ions.

Provided the stripping procedure described above is performed with a stoichiometric amount of acid, the D2EHDTPA will be present in the neutral (acidic) form, while BuPhen will exist in the freebase form. As a result, losses to the aqueous phase during stripping will be kept at a minimum. Regeneration of the extractant can be achieved by saponifying the organic phase with solid sodium hydroxide, as was done when preparing D2EHDTPA stock solutions. Alternatively, this could be achieved using a recirculating stream of sodium hydroxide, which is maintained at high pH as makeup for the consumption of sodium hydroxide by the extraction and stripping cycle. Recirculation prevents the continual loss of D2EHDTPA to a solution with a low concentration of dissolved salts. Addition of solid base to the brine is not recommended, as this can cause precipitation to occur during extraction.

### Lithium sequestration from synthetic brines

Finally, the full extraction and stripping process was tested on a simulated geothermal brine solution. The synthetic brine had a composition based on that reported for the Suho-Tungusskoe brine deposit, located in the Krasnoyarsk region in Russia (Table 1).^48^ This brine was chosen because of its challenging, high calcium matrix (58.8 g L^−1^), with only a relatively low concentration of lithium (220 ppm). Chloride salts were used to prepare the simulated brine. Because of the high required selectivity, a high concentration of BuPhen was used (1.00 mol L^−1^). The concentration of saponified D2EHDTPA was chosen to match that of lithium in the brine (0.032 mol L^−1^). The extractants were diluted in *n*-dodecane, modified with 5 vol% of 1-octanol and 20 vol% of 2-octanol. After extraction, the loaded organic phase was stripped with 32 mmol L^−1^ of HCl. The extraction and stripping process was performed in quadruplicate, and the resulting average values are given in Table 1, along with the associated standard errors. The distribution ratio and %*E* of lithium was determined by analyzing the brine before and after extraction. The extraction parameters of other elements were determined from their concentrations in the stripping solution.

**Table 1:** Extraction stripping results from a synthetic brine using 0.032 mmol L^−1^ of saponified thioD2EHPA and 1.00 mol L^−1^ of BuPhen in *n*-dodecane, modified by 5 vol% 1-octanol and 20 vol% 2-octanol. Phase volume ratio: 1 : 1, equilibrium temperature: 25 °C

Element	Conc. in feed (g L^−1^)	Conc. in strip (ppm)	%*E*	*D*	*α* (lithium to element)
Li	0.22	139	67.9 ± 0.6	2.12 ± 0.06	1
Na	46.8	174	0.341 ± 0.003	3.42 × 10^−3^^a^	620 ± 18
K	23.1	18	0.069 ± 0.004	(6.9 ± 0.4) × 10^−4^	3100 ± 200
Mg	9.5	38	0.36 ± 0.01	(3.6 ± 0.1) × 10^−3^	600 ± 10
Ca	58.8	62	0.093 ± 0.003	(9.3 ± 0.3) × 10^−4^	2300 ± 8

a Standard error <1%.

A %*E* of 68% was obtained for lithium. For all other metals, the %*E* was below 0.5%. The purity of lithium in the stripping solution was 64 mol%. The impurity ratio, defined as the ratio of the total mole fractions of the impurities in the product with respect to the feed, was 0.011. The separation factors were calculated to be 620 ± 20 for *α*_Li/Na_, 3100 ± 200 for *α*_Li/K_, 596 ± 9 for *α*_Li/Mg_ and 2290 ± 80 for *α*_Li/Ca_ (Table 1). The high values of the separation factors between lithium and the alkaline earth metals in part result from the high concentration of BuPhen on one hand, but also from the high concentrations of salts in the feed on the other hand. Just as eqn (9) describes a relationship between the separation factor *α*_Li/Mg_ and the concentration of sodium in the feed, eqn (12) can analogously be derived for the relationship between *α*_Li/Mg_ and the concentration of calcium in the feed:12
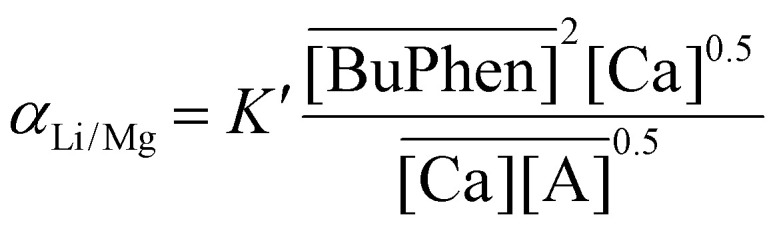


For *α*_Li/Ca_, an equivalent relationship exists with the concentration of magnesium in the feed. The parameters described above pertain to a single extraction and stripping step, but warrant further study towards a multistage countercurrent extraction process. Under these conditions, full extraction of lithium may be possible. However, these studies lie outside the scope of the present work.

The solubility of the extractants in the brine under extraction conditions was estimated using quantitative ^1^H NMR spectroscopy (full procedure given in ESI, page S7†). An organic phase consisting of 50 mmol L^−1^ D2EHDTPA, 250 mmol L^−1^ BuPhen, 2.5 vol% 1-octanol and 20 vol% 2-octanol in *n*-dodecane was equilibrated with the aforementioned synthetic brine after loading with lithium. The concentration of D2EHDTPA in the aqueous phase was below the detection limit of the measurement. For BuPhen, an aqueous concentration of 0.011 mmol L^−1^ (3.2 mg L^−1^) was obtained, a value very close to the estimated detection limit (0.003 mmol L^−1^). While the charged D2EHDTPA anion may be expected to be more water-soluble than BuPhen, the principle of charge neutrality precluded the anion from being transferred to the aqueous phase without the transfer of the lipophilic [Li(BuPhen)_2_]^+^ cation. The aqueous concentration of BuPhen can be expected to be roughly proportional to the concentration of free BuPhen in the organic phase. In this case, the free organic BuPhen concentration is approximately 150 mmol L^−1^, affording a partition coefficient of 1.4 × 10^4^.

### Comparison with other technologies for direct lithium extraction

The solvent for direct lithium extraction presented in this study is especially advantageous for recovery of lithium from brines with very high Ca/Li or Mg/Li ratios (Table 2). These cannot be treated by conventional methods, because the high reagent consumption and the lithium losses will render lithium recovery economically feasible. Geothermal brines are notable examples hereof, such as the aforementioned calcium-rich brines of Tungusskoe brine deposit in the Russian Krasnoyarsk region.^48^ Another example is the Salar de Uyuni in Bolivia, that contains relatively low concentrations of lithium (700 to 900 ppm), but high concentrations of magnesium (10 to 18 g L^−1^) and sulfate.^49^ In conventional methods, magnesium and calcium have to be removed prior to precipitation of lithium as Li_2_CO_3_, or prior to solvent extraction of lithium with Cyanex 936P or a similar extractant. Magnesium is typically removed as Mg(OH)_2_ by addition of slaked lime, while calcium is precipitated as CaCO_3_ using soda ash. Addition of slaked lime to a sulfate-rich brine will also precipitate large volumes of gypsum (CaSO_4_·2H_2_O). Large volumes of solid waste are generated as a result. Moreover, a significant fraction of the lithium (up to 50% or even more) will be lost during solid/liquid separation, either in the liquid entrapped in the solid or by adsorption to the solid.^3^ Large amounts of slaked lime and soda ash are consumed and these chemicals have a large CO_2_ footprint. Due to the high selectivity for extraction of lithium over calcium and magnesium of the solvent presented in this study, the necessity of removing these metals prior to extraction of lithium is obviated.

**Table 2:** Comparison of the state-of-the-art evaporation method and the DLE method presented in this work

	Evaporation method	This method
Water consumption (brine to LiCl)	Approx. 100–800 m^3^ of evaporative losses per ton of Li_2_CO_3_ equivalent (over 90% of original brine water)^50^	No theoretical consumption
Reagent use (brine to LiCl)	1 eq. of lime with respect to Mg and sulfate (large excess with respect to Li)	1 eq. of NaOH and 1 eq. of HCl with respect to Li
Waste production	Large quantities of Mg(OH)_2_ and gypsum (all Mg and sulfate from brine)	1 eq. of NaCl with respect to Li
	Over 90% of total dissolved salts are crystallized as waste^50^	
Li recovery yield	Approx. 50% ^3^	Approx. 68% in one contact (higher possible using multistage solvent extraction)
Plant footprint	Very large (evaporation and precipitation ponds)	Limited (mixer-settlers)

The TBP/FeCl_3_ system, which is also selective for lithium over magnesium and calcium, is more difficult to strip than the system presented in this study. Concentrated HCl solutions of up to 6 mol L^−1^ and extreme phase ratios are required to prevent the loss of FeCl_3_ during stripping.^31^ The system described herein can be stripped using stoichiometric solutions of HCl. Another advantage of our solvent is that is does not contain fluorinated molecules, unlike several other lithium-selective extractants. Fluorinated compounds must be avoided because they are persistent (“forever chemicals”). Furthermore, bis(2-ethylhexyl)dithiophosphoric acid (D2EHDTPA) and even 2,9-dibutyl-1,10-phenanthroline are easier to synthesize than macrocyclic extractants such as crown ethers and calix[4]pyrroles, which typically require complex multistep procedures with low yields. As a result, the mass of waste generated during the synthesis of the extractants used in this study will be smaller (*i.e.* a smaller *E*-factor).

Compared to solid adsorbents or ion exchangers, solvent extraction systems feature significantly more rapid kinetics of lithium uptake, and far higher capacities for metal loading. This results in an improved time space yield. Furthermore, the purified solutions yielded by adsorbents are dilute, requiring concentration by boiling in order to allow precipitation of lithium.^11,12^ By contrast, solvent extraction allows the concentration of the feed by altering the phase ratio during extraction and stripping.

## Conclusion

The synergistic mixture of saponified bis(2-ethylhexyl)dithiophosphoric acid (D2EHDTPA) and 2,9-dibutyl-1,10-phenanthroline (BuPhen) was found to selectively extract lithium over other alkali and alkaline earth ions. The soft sulfur donor atoms make D2EHDTPA an ideal liquid ion exchanger, allowing BuPhen to confer selectivity to the system by forming a 2 : 1 complex with lithium. The selectivity for lithium improves with lower concentrations of D2EHDTPA, higher concentrations of BuPhen, lower temperatures and higher concentrations of alkali and alkaline earth salts in the brine. Stripping can be achieved with stoichiometric amounts of strong acids, yielding a neutral salt solution that is highly enriched in lithium and ready for further downstream purification. A test using a synthetic geothermal brine yielded a percentage extraction of 68% for lithium, and separation factors of 620 over sodium, 3100 over potassium, 600 over magnesium and 2300 over calcium. Full extraction of lithium is not possible in one stage, but the distribution ratio and separation factors are sufficiently high for design of a multistage countercurrent solvent extraction process. Compared to the state-of-the-art process for treatment of lithium brines, the proposed DLE process obviates the need for brine evaporation, reducing both water consumption and the footprint of the plant. Furthermore, the alkaline earth elements no longer need to be precipitated, drastically curbing reagent use and waste production. Hence, the proposed process offers opportunities to significantly improve the sustainability of brine lithium production, while unlocking geothermal brines as potential future lithium sources.

## Data availability

The data supporting this article have been included as part of the ESI.†

## Conflicts of interest

There are no conflicts to declare.

## Supplementary Material

GC-027-D4GC04760E-s001
