# Supplementary material for: Selective extraction of lithium over alkali and alkaline earth ions by synergistic solvent extraction
Source: Green Chem. 2024 Dec 20;27(4):1194–205. doi: 10.1039/d4gc04760e (PMC11694342; doi:10.1039/d4gc04760e)
Supplement: GC-027-D4GC04760E-s001 [file GC-027-D4GC04760E-s001.pdf]

Supplementary Information (SI) for Green Chemistry.
This journal is © The Royal Society of Chemistry 2024

Electronic Supplementary Information (ESI)

Selective Direct Extraction of Lithium over Alkali and Alkaline Earth Ions by Synergistic Solvent Extraction

Stijn Raiguel[†*], Laura Van Bogaert[†], Tim Balcaen[†] and Koen Binnemans[†]

[†] KU Leuven, Department of Chemistry, Celestijnenlaan 200F, P.O. box 2404, B-3001 Leuven, Belgium.

*Corresponding author:

Email: stijn.raiguel@kuleuven.be

Table of contents

Chemicals and reagents S3

Synthesis of extractants S4

Procedure for solvent extraction S7

Instrumentation S8

^{13}C NMR of loaded organic phase S9

^{31}P NMR of loaded organic phase S10

Van ‘t Hoff study of exchange equilibrium: details on regression and *t*-test S11

Spectra of synthesized compounds S12

References S16

Chemicals and reagents

Dimethyl sulfoxide-d_6 (99.9 atom% D), phosphorus pentasulfide (99%), 1-butanol (Emsure®), *n*-butyllithium (2.5 M in hexanes), 1,10-phenanthroline (≥99.9%), tetrahydrofuran (≥99.9% for HPLC), nitric acid concentrate (0.1 M, eluent for IC), pyridine-2,6-dicarboxylic acid (≥99.5%, suitable for IC), lithium chloride (99%), magnesium chloride hexahydrate (Emsure®) and 2-octanol (97%) were purchased from Merck Life Science BV (Overijse, Belgium). Heptane (laboratory reagent grade), sodium hydrogen carbonate (analytical reagent grade), HCl (37%, analytical reagent grade), dichloromethane (≥99.8%), sodium chloride (99.5%), sodium hydroxide (analytical reagent grade), deuterium oxide (for NMR, 99.8 atom% D) and orthophosphoric acid (analytical reagent grade) were obtained from Fischer Scientific BVBA (Merelbeke, Belgium). Toluene (ACS reagent), sodium carbonate (Ph. Eur.) and calcium chloride dihydrate (AnalR NormaPur®) were acquired from VWR International BV (Leuven, Belgium). Lithium, sodium, potassium, rubidium, magnesium and calcium standard solutions for IC (1000 ppm, Ion HIQU) and potassium chloride (a.r.) were purchased from Chem-Lab NV (Zedelgem, Belgium). Manganese(IV)oxide (99%, -10 mesh), bis(2-ethylhexyl) hydrogen phosphate (95%), rubidium chloride (99.8%), lithium hydroxide anhydrous (99.8%), trimethyl phosphate (p.a.) and 1-octanol (99%, pure) were obtained from Acros Organics BV (Geel, Belgium). *n*-Dodecane was acquired from TCI (Zwijndrecht, Belgium). Cyanex® 301 was purchased from Solvay NV (Brussels, Belgium). Lithium bis(trifluoromethylsulfonyl)imide (99%) was obtained from IoLiTec GmbH (Heilbronn, Germany).

Tetrahydrofuran was dried prior to use using a MBraun MB SPS-800 solvent purifier. All other chemicals were used as received. Ultrapure water was generated using a Millipore Milli-Q Reference® system, purified to a TOC of < 2 ppb and a resistivity of 18.2 MΩ cm.

Synthesis of extractants

Bis(2-ethylhexyl)dithiophosphoric acid

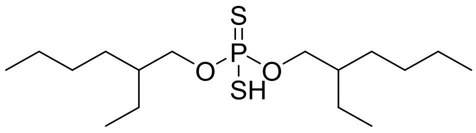

Bis(2-ethylhexyl)dithiophosphoric acid (D2EHDTPA, Fig. 2) was prepared by reaction between phosphorus pentasulfide (P_4S_{10}) and 2-ethylhexanol, based on the procedure reported by Aswath *et al.*[1] A flask was charged with 6.40 g (14.4 mmol, 0.15 eq.) of P_4S_{10} and 40 mL of toluene. A 20 g portion of 3 Å molecular sieves and 15 mL of 2-ethylhexanol (95.5 mmol, 1.0 eq) were added. The flask was then flushed with nitrogen gas and equipped with a NaOH gas trap to neutralize H_2S effervescence. The mixture was stirred at 100 °C overnight, during which time the color changed to dark green. The reaction mixture was subsequently allowed to cool and filtered under vacuum. The residue was washed with 40 mL of heptane. Next, 10 mL of 1-butanol was added to the filtrate (to aid in phase separation). The reaction mixture was stirred with 50 mL of saturated sodium hydrogen carbonate solution for 2 hours, subsequently with 100 mL of 1 mol L^{-1} hydrochloric acid for 1 hour, then with 100 mL of 1 mol L^{-1} of sodium carbonate solution for 2 hours, and finally with 100 mL of 0.5 mol L^{-1} hydrochloric acid for 20 min. The color changed from dark green to practically colorless during the sodium carbonate wash. Finally, the organic phase was dried over magnesium sulfate and concentrated under reduced pressure, to afford the title compound as a pale green oil in a yield of 88% (14.92 g, 42.1 mmol).

^{1}H NMR ($CDCl_3$, 400 MHz, ppm): 4.07 (4 H, m, 2 C**H**$_2$-O), 3.09 (1 H, brd. s, S**H**), 1.65 (2 H, m, 2 C**H**), 1.25-1.51 (16 H, m, 8 C**H**$_2$), 0.83-1.00 (12 H, 2 t, 4 C**H**$_3$).

^{13}C NMR ($CDCl_3$, 100 MHz, ppm): 70.47 & 70.40 (O-**C**H_2, 2 diastereomers), 39.74 & 39.75 (**C**H, 2 diastereomers), 29.99 (**C**H_2), 28.89 (**C**H_2), 23.40 (**C**H_2), 22.95 (**C**H_2), 14.05 (**C**H_3), 10.95 (**C**H_3).

^{31}P NMR ($CDCl_3$, 162 MHz, ppm): 85.71.

MS (ASAP-): *m/z* calculated for $C_{16}H_{34}O_2PS_2^-$: 353.2 [*M*-H]$^-$; found: 353.2 [*M*-H]$^-$.

Small amounts of mono-2-ethylhexyldithiophosphoric acid may be formed during the reaction (due to the presence of trace amounts of water), which can be removed by the sodium carbonate washing step. The presence of these impurities may result in the formation of a gel during solvent extraction, in particular in the presence of high concentrations of alkaline earth metals.

2,9-Dibutyl-1,10-phenanthroline

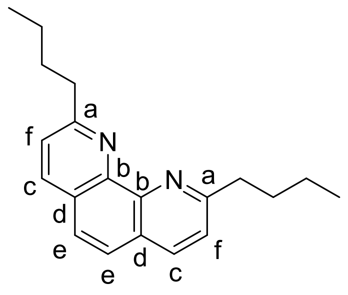

2,9-Dibutyl-1,10-phenanthroline was synthesized by addition of *n*-butyllithium to 1,10-phenanthroline, followed by oxidative rearomatization. This procedure is based on that reported by Yang and Nakano.[2] A 3.60 g portion of anhydrous 1,10-phenanthroline (20 mmol, 1.0 eq.) was combined with 100 mL of dry tetrahydrofuran (THF) in a flame-dried flask under inert (argon) atmosphere. The solution was cooled to 0 °C using a cryostat, and 24 mL of a 2.5 mol L^{-1} solution of *n*-butyllithium in hexanes (60 mmol, 3.0 eq.) was added over 30 minutes, causing an immediate dark red coloration of the reaction mixture. The mixture was allowed to stir

for 24 h at 0 °C. Subsequently, the mixture was quenched by the slow addition of water (40 mL). The aqueous phase was discarded. Solvents were removed from the organic layer under reduced pressure. To the resulting orange oil, 100 mL of dichloromethane (DCM) was added, along with 8.69 g of fine-grained manganese dioxide (100 mmol, 5 eq.). The reaction mixture was then stirred overnight at room temperature. Subsequently, the mixture was filtered. The filtrate was concentrated under reduced pressure. The crude product (5.60 g, ^{1}H NMR given in Fig. S8) was recrystallized from 100 mL of heptane at 0 °C. A second crop of crystals was obtained by reducing the volume to 30 mL after the first crystallization step, and again cooling the solution to 0 °C. A third crop was collected after reducing the volume to 10 mL and once again cooling to 0 °C. Combining all three batches of crystals afforded 3.77 g (12.9 mmol) of the desired compound as yellow crystals in a yield of 64%.

^{1}H NMR ($CDCl_3$, 400 MHz, ppm): 8.15 (2 H, d, ^{3}J = 8.0 Hz, C**H** c), 7.71 (2 H, s, C**H** e), 7.53 (2H, d, ^{3}J = 8.0 Hz, C**H** f), 3.24 (4 H, m, α-C**H**$_2$), 1.94 (4 H, m, β-C**H**$_2$), 1.55 (4 H, sext, ^{3}J = 7.5 Hz, γ-C**H**$_2$), 1.03 (6 H, t, ^{3}J = 7.5 Hz, C**H**$_3$).

^{13}C NMR ($CDCl_3$, 100 MHz, ppm): 163.29 (**C**H a), 145.51 (**C**H b), 136.15 (**C**H c), 127.07 (**C**H d), 125.44 (**C**H e), 122.32 (**C**H f), 39.28 (**C**H$_2$), 31.87 (**C**H$_2$), 22.97 (**C**H$_2$), 14.09 (**C**H$_3$).

MS (ASAP+): *m/z* calculated for $C_{20}H_{25}N_2^{+}$: 293.2 $[M+H]^{+}$; found: 293.2 $[M+H]^{+}$, 250.1 (McLafferty product).

Note that it is imperative to use fine-mesh (< 10 mesh) MnO_2 for this procedure. An attempt at using coarse crystalline MnO_2 was not successful.

Procedure for solvent extraction

Solvent extraction experiments were carried out in 4 mL vials. The organic phase was prepared by diluting stock solutions of D2EHDTPA and BuPhen with *n*-dodecane, 1-octanol and 2-octanol. The D2EHDTPA stock solution was saponified by stirring overnight with 1 eq. of solid sodium hydroxide. Phase separation was expedited by centrifugation (Heraeus Labofuge 200). The concentration of D2EHDTPA in the organic phase was chosen to be stoichiometric with the amount of lithium in the aqueous phase, in order to improve separation through saturation effects. An aliquot of the solvent was then contacted with a desired volume of the aqueous feed solution for 1 h by shaking in the horizontal position at 2000 rpm. The equilibrium temperature was 25 °C and the phase volume ratio was 1:1, unless stated otherwise. Phase separation was again expedited by centrifugation. The aqueous phase was then analyzed using ion chromatography (IC). If multiple replicates were measured, errors are reported as the standard error on the mean.

qNMR procedure

A 2000 μL aliquot of synthetic brine was contacted with 500 μL of the desired organic phase, preloaded with lithium from an identical brine. The phases were equilibrated by shaking at 2500 rpm for 2 hours. A 1600 μL sample of the aqueous phase was subsequently extracted with 800 μL of chloroform-*d*. A 400 μL sample of the chloroform layer was then combined with 100 μL of a standard solution containing 2.294 mmol L^{-1} of trimethyl phosphate in chloroform-*d*. A ^{1}H NMR spectrum was recorded (400 MHz, 16 scans, d1 = 30 s), and the signal of trimethyl phosphate was used as internal reference. The phenylic protons of BuPhen were used for integration. No characteristic signals of D2EHDTPA were detected in the spectrum.

Instrumentation

High-field ^1H, ^{13}C, ^{31}P, ^7Li, ^1H-^{13}C HSQC-DEPT nuclear magnetic resonance (NMR) spectra were recorded on a on a Bruker Avance III HD 400 spectrometer with a Bruker Ascend™ 400 magnet system (^1H basic frequency of 400.17 MHz) and a 5 mm PABBO BB/19F-1H/D probe with z-gradients, or on a Bruker Avance II+ 600 spectrometer with a Bruker 600 UltraShield™ magnet system (^1H basic frequency of 600.13 MHz) and a 5 mm PABBO BB-1H/D probe with z-gradients. ^{13}C- and ^{31}P-detected experiments were ^1H-decoupled using inverse-gated broadband decoupling. All samples were dissolved in chloroform-d, or measured in neat condition in tandem with a DMSO-d_6 sample placed in a glass insert. Data were recorded at room temperature using Bruker TopSpin 3.6.x and processed and analyzed using Spinworks 4.2.8. ^{13}C data were calibrated using DMSO-d_6 as internal calibration reference (39.52 ppm). The δ-values are expressed in parts per million (ppm). The following acronyms were used: s (singlet), d (doublet), t (triplet), sext (sextet), m (multiplet), brd (broadened). Recycle delay times were not optimized.

Mass spectra were recorded on a Waters Radian ASAP instrument. The flow gas (N_2) temperature was set to 450 °C. Spectra were analyzed using the MassLynx 4.2 SCN software package

Ion chromatography was carried out on a Shimadzu device consisting of a of a CBM-40 system controller, DGU-403 degassing unit, LC-20Ai pump, SIL20A autosampler, CTO-40C column oven and CDD-10A vp conductivity detector. A Metrohm Metrosep C4 column was used. The eluent consisted of 1.7 mmol L^{-1} nitric acid and 0.7 mmol L^{-1} pyridine-2,6-dicarboxylic acid in ultrapure water. The oven temperature was set at 25 °C and the flow rate of the eluent was 0.9 mL min^{-1}. Samples were diluted to obtain concentrations in the 1–200 ppm range for every analyte. The injection volume was varied between 3 and 10 µL in order to obtain both sufficient peak

intensity and resolution. Calibration was performed using an external standard solution series, to which a linear calibration curve was fitted.

^{13}C NMR of loaded organic phase

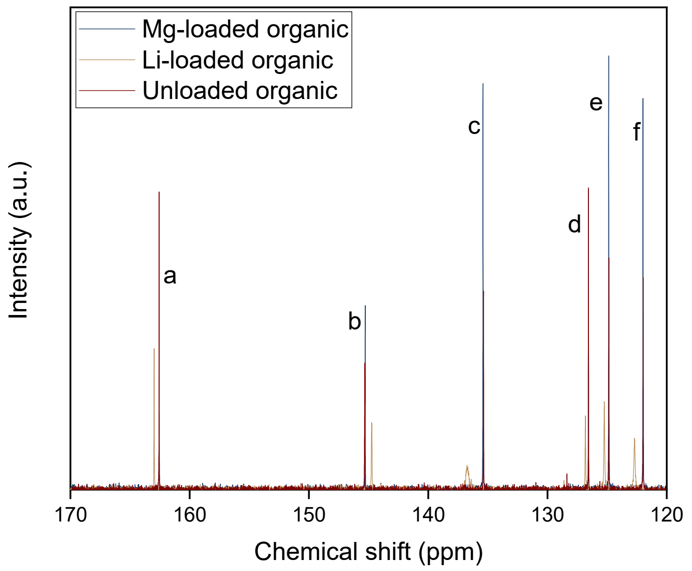

Fig. S1: Aromatic region of ^{13}C NMR spectra of 0.05 mol L^{-1} D2EHDTPA and 0.100 mol L^{-1} BuPhen in n-dodecane with 2.5 vol% 1-octanol and 20 vol% 2-octanol loaded with Mg^{2+} vs. Li^{+}, compared to unloaded 0.100 mol L^{-1} BuPhen in n-dodecane with 2.5 vol% 1-octanol and 20 vol% 2-octanol. Spectra are referenced to the n-dodecane methyl signal (13.61 ppm). Assignments given in on Page S5.

^{31}P NMR of loaded organic phase

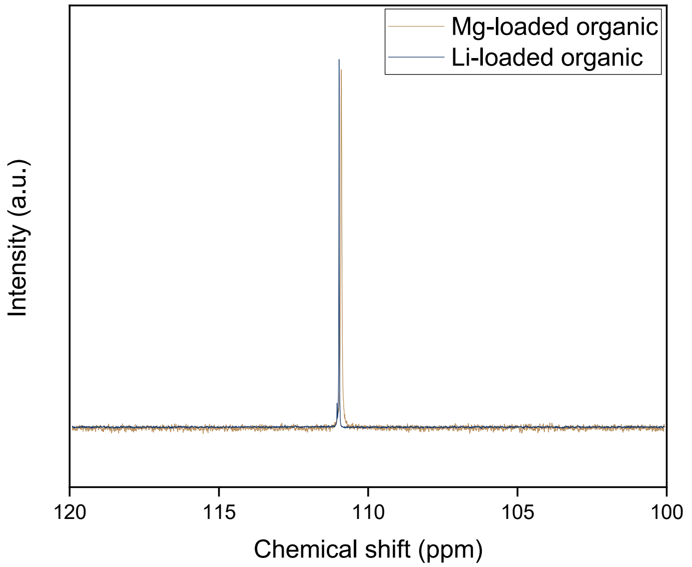

Fig. S2: Thiophosphate region of ^{31}P NMR spectra of 0.05 mol L^{-1} D2EHDTPA and 0.100 mol L^{-1} BuPhen in n-dodecane with 2.5 vol% 1-octanol and 20 vol% 2-octanol loaded with Mg^{2+} vs. Li^{+}. Spectra are referenced to 10 vol% H_3PO_4 in D_2O (0.00 ppm).

Van ’t Hoff study of exchange equilibrium: regression details and paired *t*-test

Table S1: Details of linear regression (ordinary least-squares estimates) of $ln(K_{eq})$ as a function of the reciprocal absolute temperature reported as mean ± standard error on the mean. Data was fitted using Origin 2018b.

	**Li/Mg**	**Li/Ca**
Slope	2918 ± 97	2966 ± 139
Intercept	-1.04 ± 0.31	-0.96 ± 0.44
R^2	0.997	0.993

Ordinary least-squares linear regression analysis was performed using Origin 2018b software. Estimates of slope and intercept, combined with coefficient of determination are given in Table S1. The parametric, two-tailed paired *t*-test was performed using JMP® Pro 17.0.0 software. Pairing of the data by temperature was justified on the basis of simultaneous within-sample measurements of Ca^{2+} and Mg^{2+} concentrations. Assumptions of homoscedasticity and normality are found justifiable based on the residual plot of a multivariate linear model of the form:

$$ln(K) = \beta_0 + \beta_1 * \left(\frac{1}{T}\right) + \beta_2 * (Metal) + \gamma * (Sample) + \varepsilon \quad \text{Eq. S1}$$

In Eq. S1, β_0 refers to the entropy value, the β_1 refers to the enthalpy value and β_2 refers to the effect of the metal on the intercept, which are the fixed effects of the model. γ refers to the random effect attributed to the sample after pairing and ε refers to the model error.

NMR spectra

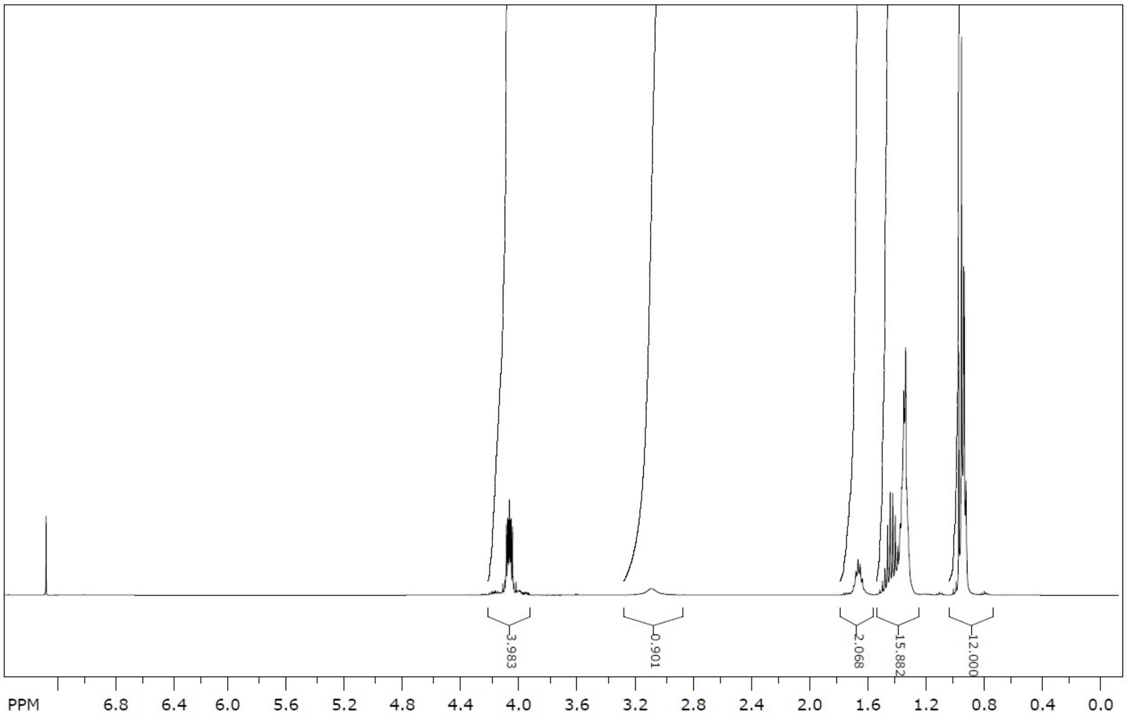

Fig. S3: ^{1}H NMR spectrum of bis(2-ethylhexyl)dithiophosphoric acid (400 MHz, $CDCl_3$).

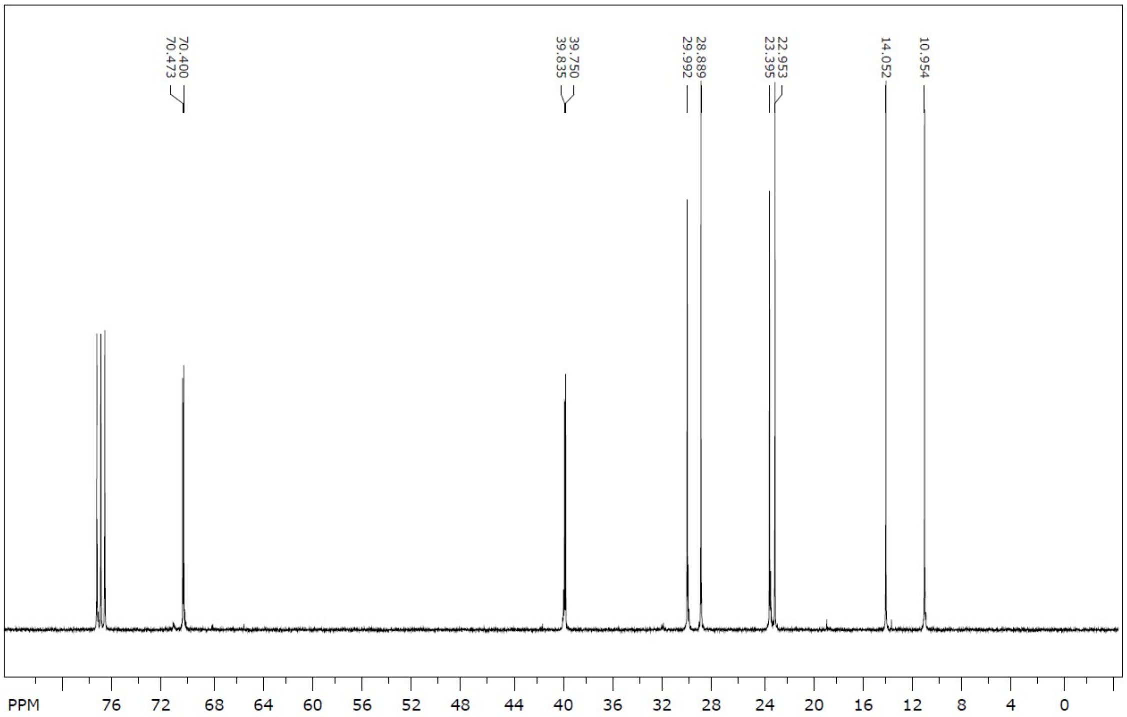

Fig. S4: ^{13}C NMR spectrum of bis(2-ethylhexyl)dithiophosphoric acid (100 MHz, $CDCl_3$).

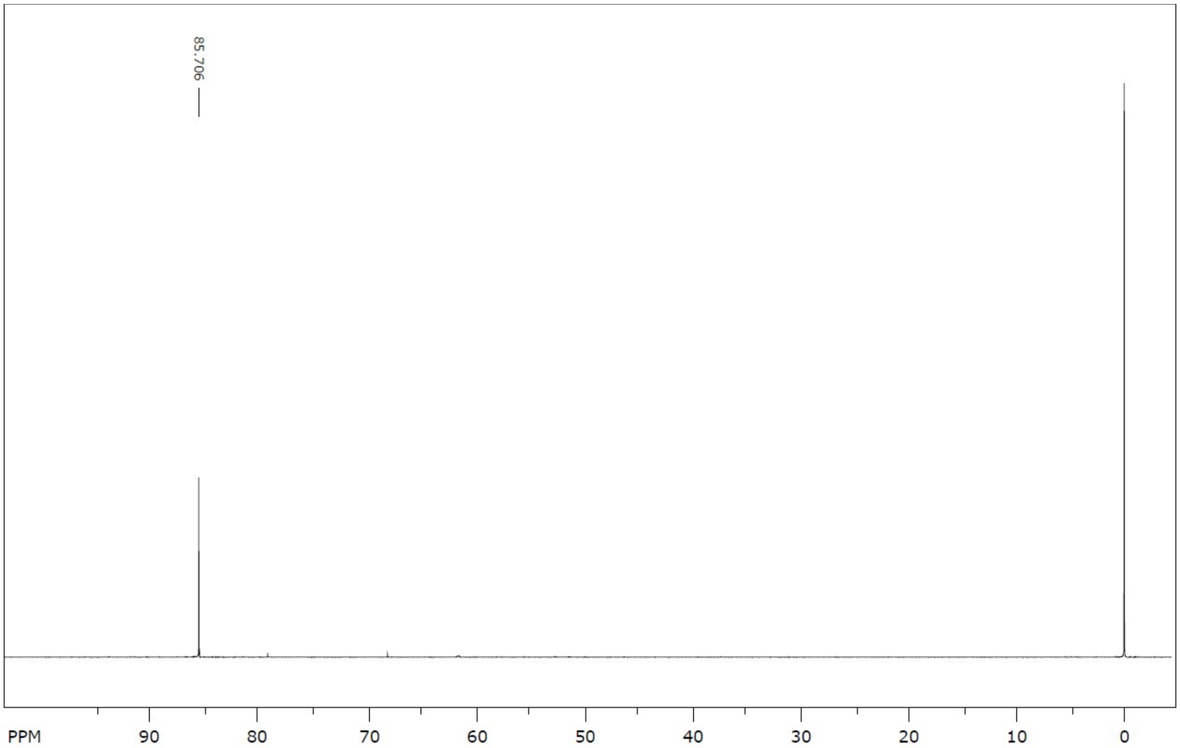

Fig. S5: ^{31}P NMR spectrum of bis(2-ethylhexyl)dithiophsphoric acid (100 MHz, $CDCl_3$). External reference: 10 vol% H_3PO_4 in D_2O (0.00 ppm).

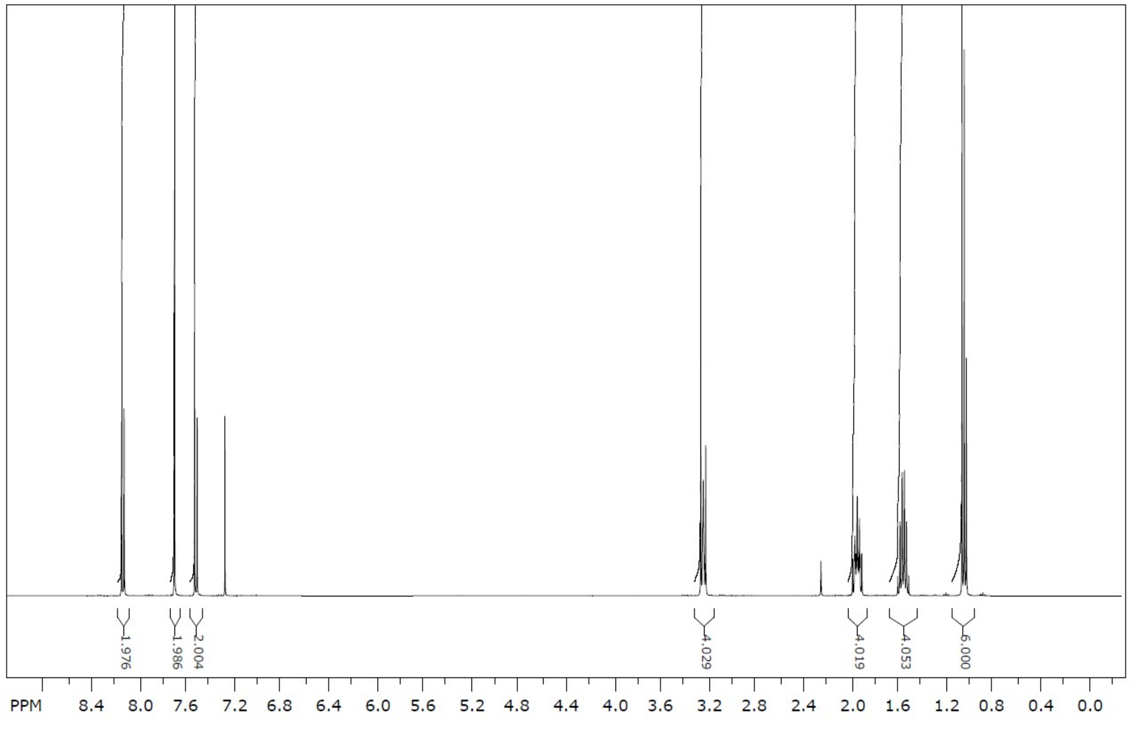

Fig. S6: ^{1}H NMR spectrum of 2,9-dibutyl-1,10-phenanthroline (400 MHz, $CDCl_3$). Trace water observed at approx.. 2.2 ppm.

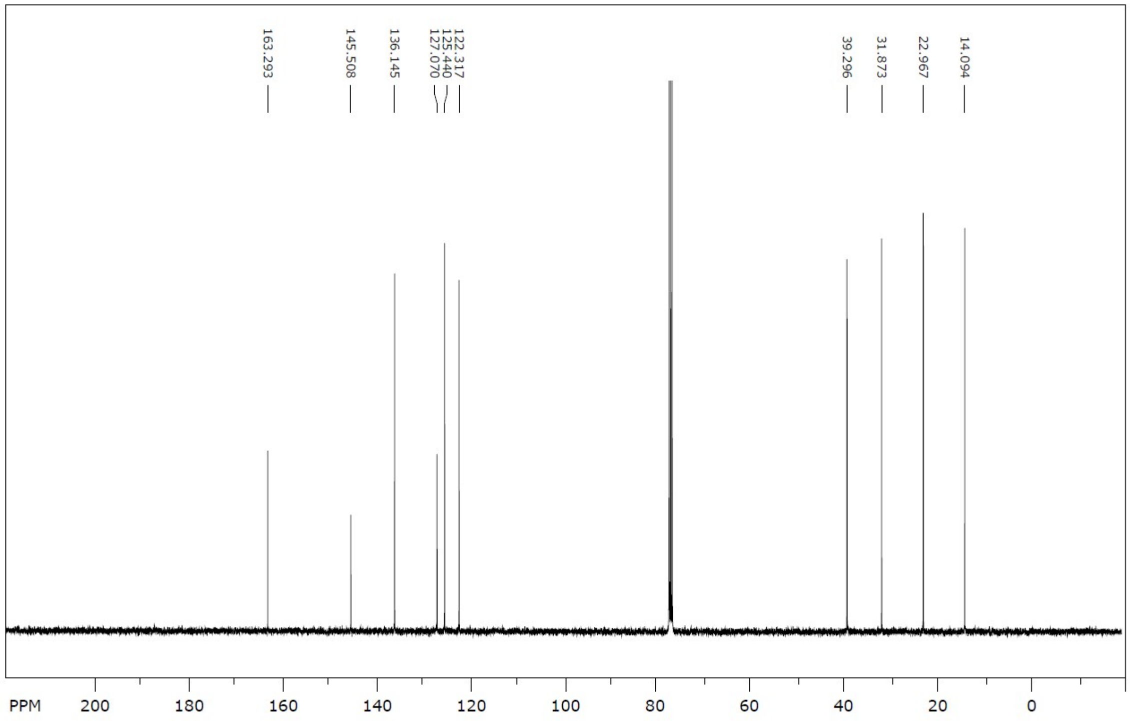

Fig. S7: ^{13}C NMR spectrum of 2,9-dibutyl-1,10-phenanthroline (100 MHz, $CDCl_3$).

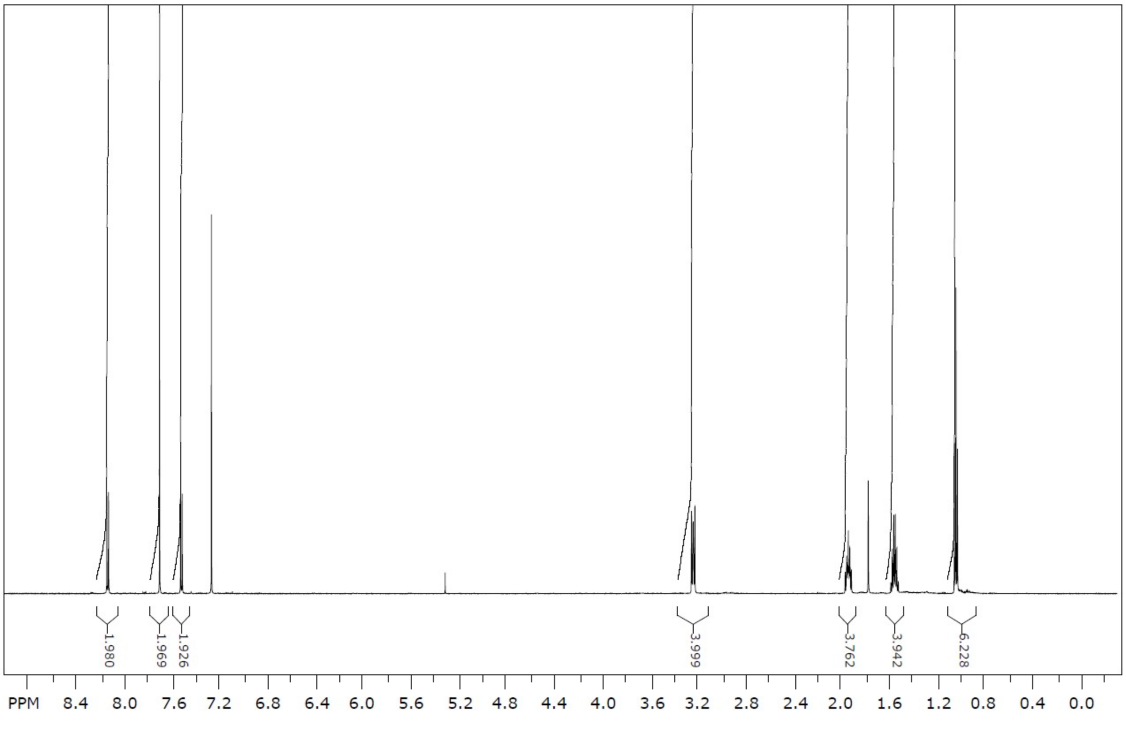

Fig. S8: ^{1}H NMR spectrum of crude 2,9-dibutyl-1,10-phenanthroline prior to recrystallization. Residual DCM (approx. 5.3 ppm) and water (approx. 1.8 ppm) are visible. Spectrum recorded on a Bruker Avance II+ operating at 600 MHz in $CDCl_3$.

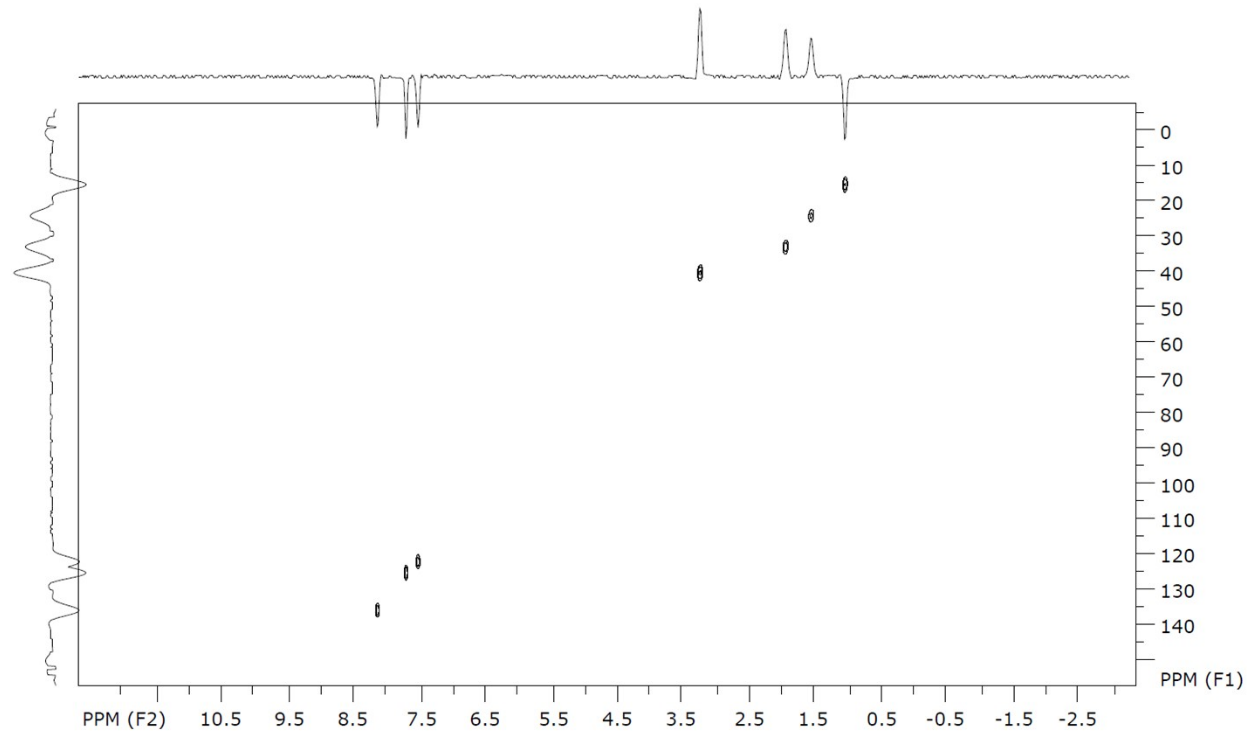

Fig. S9: ^{1}H-^{13}C HSQC-DEPT NMR spectrum of 2,9-dibutyl-1,10-phenanthroline (600 MHz, $CDCl_3$).

References

1 *US Pat.*, US20130296598A1, 2013.
2 W. Yang and T. Nakano, *Chem. Commun.*, 2015, **51**, 17269–17272.
